# AnaToc75 (Alr2269) mediates calcium uptake across the outer membrane in *Anabaena* sp. PCC 7120

**DOI:** 10.1128/jb.00291-25

**Published:** 2025-09-18

**Authors:** Xiaoying Jiang, Hong Gao, Yanling Dong, Jianxin Tang, Shuiling Ji, Xudong Xu

**Affiliations:** 1 Institute of Hydrobiology, Chinese Academy of Sciences 53021 , Wuhan, Hubei, China; 2 University of Chinese Academy of Sciences 74519, Beijing, China; 3 Key Laboratory of Pesticide & Chemical Biology of Ministry of Education, Hubei Key Laboratory of Genetic Regulation and Integrative Biology, School of Life Sciences, Central China Normal University 12446, Wuhan, Hubei, China; Queen Mary University of London, London, United Kingdom

**Keywords:** Ca^2+^ uptake, outer membrane, Toc75 homolog, cyanobacteria

## Abstract

It has been a long-standing notion that cyanobacterial Omp85 proteins are essential because of their role in insertion of β-barrel proteins into the outer membrane (OM). Alr2269 from *Anabaena* sp. PCC 7120 is such an Omp85 protein, structurally related to the Toc75 channel of the translocon at the outer envelope membrane of chloroplasts but is actually non-essential to *Anabaena* under calcium-replete conditions. In a completely segregated *alr2269*-null mutant, a predicted S-layer protein, All3983, and many β-barrel proteins in the OM were upregulated compared with the wild type. By removal of each component from the medium, Ca^2+^ deficiency was identified to be the key environmental signal for upregulation of P*_all3983-_luxAB* in the mutant. Assays of ^45^Ca^2+^ uptake in the wild type and the mutant indicated that Alr2269 is a relatively specific channel for transport of Ca^2+^ across the OM in low-Ca^2+^ medium. Accordingly, inactivation of *alr2269* greatly reduced the growth under Ca^2+^-limiting conditions, while the complemented strain grew as the wild type. In low-Mg^2+^ medium, however, the *alr2269* mutant showed only a slight difference in growth from the wild type. These results establish Alr2269 (AnaToc75) as the main channel for Ca^2+^ uptake across the OM of *Anabaena* in low-Ca^2+^ environments.

**IMPORTANCE:**

Ca^2+^ is required for photosynthesis and various cellular activities in cyanobacteria, but Ca^2+^ uptake, in particular how Ca^2+^ is transported across the outer membrane, has been barely investigated in cyanobacterial species. In this study, we found that a cyanobacterial Toc75 homolog is not necessarily essential for protein integration into the outer membrane as was thought before but instead is required for Ca^2+^ uptake in low-Ca^2+^ environments. This finding not only establishes a Ca^2+^ channel across the outer membrane of cyanobacteria but also provides an example of additional functions for Omp85 proteins.

## INTRODUCTION

Ca^2+^ is required for various biological processes, functioning as secondary messengers in cellular signaling (1, 2) and structural/catalytic cofactor for various enzymes (3–5). In cyanobacteria, Ca^2+^ plays an important role in photosynthesis (6, 7), motility (8, 9), cell differentiation (10), and stress responses (11, 12), and Ca^2+^ fluctuations synchronize cellular activities like circadian rhythms (13) and secondary metabolite production (14), crucial for ecological fitness and resource optimization. Furthermore, findings in Gram-negative bacteria (15) suggest that Ca^2+^ could play a structural role in the outer membrane of cyanobacteria as well, particularly in stabilization of the lipopolysaccharide layer and organization of S-layer proteins.

Although Ca^2+^ is so important to life activities, cytosolic free Ca^2+^ is maintained at submicromolar levels in cyanobacteria (10, 16, 17), about three orders of magnitude lower than the culture medium level, and may increase to 1–4 µM transiently in response to environmental stresses (16, 17). Similar differences between intra- and extracellular Ca^2+^ concentrations are found in other free-living bacteria (18, 19). For this reason, Ca^2+^ extrusion mechanisms (20) are much more extensively studied than uptake mechanisms (21–23). Regardless of efflux or influx, almost all Ca^2+^ transport systems hitherto reported in bacteria (24, 25), including those in cyanobacteria, are located in the cytoplasmic membrane, except the Ca^2+^ transporter across the outer membrane in *Mycobacterium tuberculosis* (26).

Alr2269 is an outer membrane protein in the heterocyst-forming cyanobacterium *Anabaena* sp. PCC 7120 (hereafter referred to as *Anabaena* 7120) and structurally related to Toc75 of the chloroplast outer envelope translocon complex (27), therefore dubbed AnaToc75. Alr2269 and homologs, such as Slr1227 (SynToc75) from *Synechocystis* sp. PCC 6803 (28, 29), belong to the Omp85 superfamily. Generally, Omp85 proteins in bacteria are involved in the insertion of β-barrel proteins into the outer membrane or translocation of proteins across the outer membrane (30). Cyanobacterial homologs of Toc75 are thought to be essential because both *alr2269* and *slr1227* mutants failed to be completely segregated (29, 31). One explanation is that they are required for integrating β-barrel proteins into the outer membrane ensuring membrane integrity and permeability (31, 32). Indeed, a partially segregated mutant of *alr2269* showed altered sensitivity to erythromycin, SDS, lysozyme, and proteinase K and enhanced permeability to sucrose and glutamate. On the other hand, SynToc75 in reconstituted liposomes exhibits solute channel properties, allowing ions and small peptides to pass through the lipid layer (28). Thus, cyanobacterial homologs of Toc75 may act as ion channels as well.

**Fig 1:**
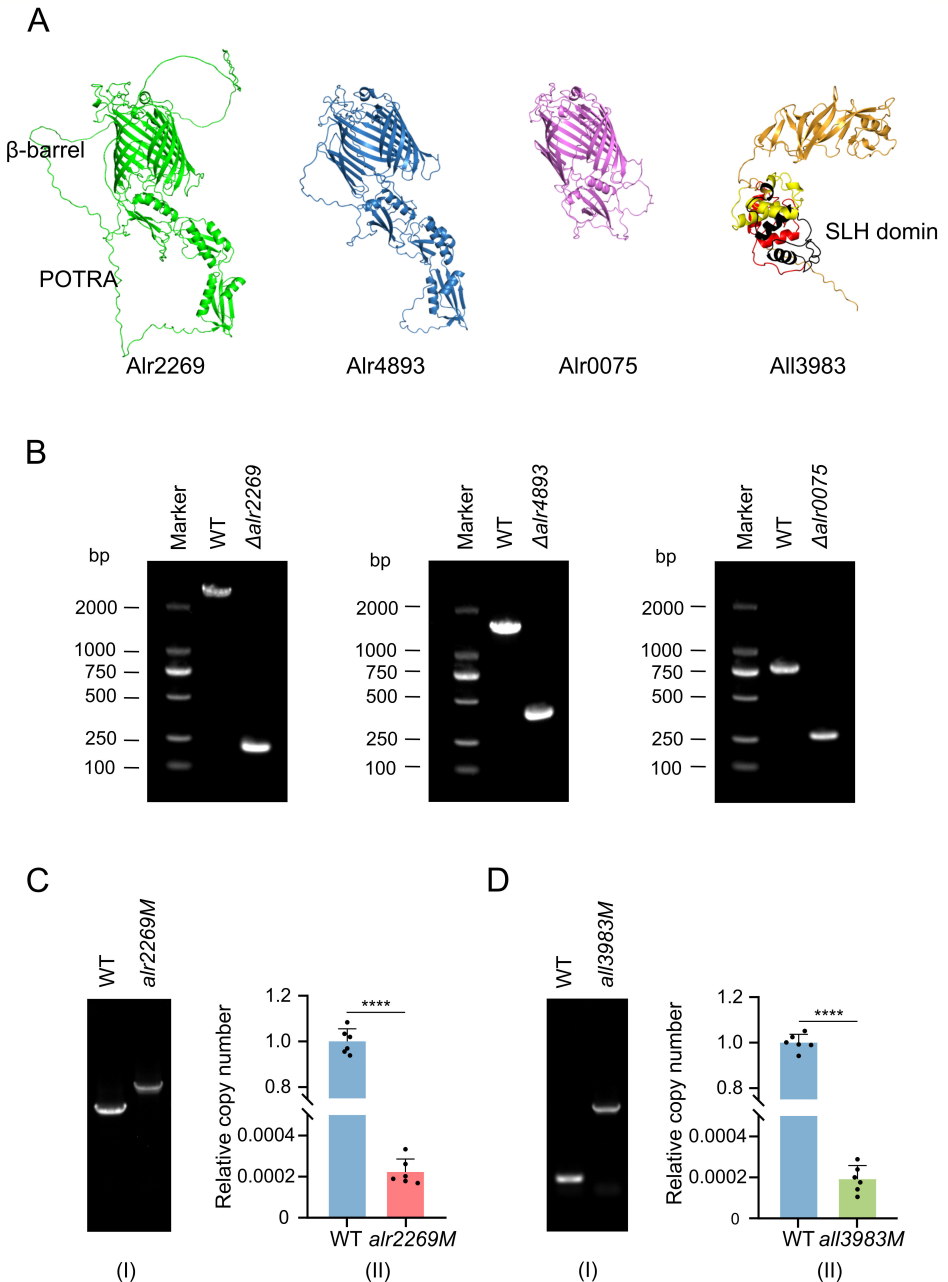
Predicted structures of Alr2269, Alr4893, Alr0075, and All3983 and the completely segregated *Anabaena* 7120 mutants of their encoding genes. PCR primers are indicated in Fig. S2 and listed in Table S1. (**A**) Predicted structures of Alr2269, Alr4893, Alr0075, and All3983. The three SLH domains (amino acid residue no. 21–84, 85–144, 146–210) are indicated in red, black, and yellow. (**B**) Electrophoretograms demonstrating PCR products amplified from genomes of *Anabaena* 7120 (WT) and the deletion mutants *Δalr2269, Δalr4893*, and *Δalr0075*. (**C and D**) PCR (I) and qPCR (II) examinations showing the complete segregation of the *alr2269M* and *all3983M* mutants. The relative copy numbers were calculated as 2^-△△^*^Ct^* values in Tables S4 and S5.

In *Anabaena* 7120, there are two other Omp85 proteins, Alr4893 and Alr0075, homologous to Alr2269 (31). These Omp85 proteins (Fig. 1A) share a conserved β-barrel-type pore structure and the N-terminal POTRA (polypeptide translocation associated) domains (33) but differ in the pore diameter (1.3, 1.2, and 1.7 nm for Alr4893, Alr0075, and Alr2269, respectively) (31) and the number of POTRA domains (three for Alr4893 and Alr2269, one for Alr0075, see Fig. 1A). Like *alr2269*, *alr4893* and *alr0075* appeared to be essential to *Anabaena* 7120 because none of their insertion mutants were completely segregated (31, 32).

The lack of completely segregated mutants of Toc75 homolog genes in cyanobacteria may greatly impede the in-depth investigation of their functions because some phenotypes could be masked by the presence of the wild-type gene. Furthermore, the mutant strains would not be stable until complete segregation. For example, partially segregated *alr2269* mutants showed very different sensitivities to lysozyme, proteinase K, SDS, and erythromycin in two independent reports (31, 32). In this study, we obtained the completely segregated *alr2269*-null mutant of *Anabaena* 7120, then we identified All3983, a predicted S-layer protein, as the Ca^2+^-responsive marker in the *alr2269* background. Ca^2+^ uptake and low Ca^2+^ growth experiments showed the role of Alr2269 in transport of Ca^2+^ across the outer membrane. Unlike *alr2269*, the other two Omp85 protein genes were not required for growth in low Ca^2+^ medium. These results established Alr2269 (AnaToc75) as an important Ca^2+^ channel under Ca^2+^-limiting conditions, showing the role of a Toc75 homolog in ion transport.

## RESULTS

### Generation and characterization of the completely segregated *alr2269* mutant

**Fig 2:**
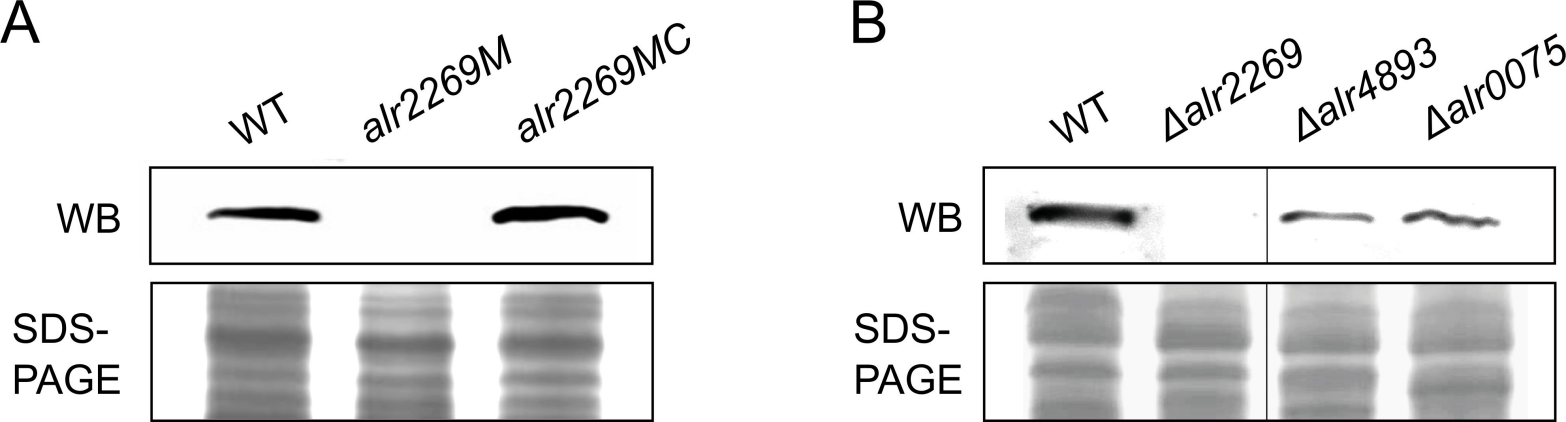
Western blot detection (WB) of Alr2269 in the wild type and mutants of *Anabaena* 7120. (**A**) Detection of Alr2269 in the wild type, *alr2269M,* and the complementation strain *alr2269MC*. (**B**) Detection of Alr2269 in the wild type, *Δalr2269, Δalr4893*, and *Δalr0075*.

Previous studies reported that all three Omp85 protein genes are essential to *Anabaena* 7120 (31, 32). However, employing the CRISPR-Cpf1-based genome editing system (34, 35), we successfully deleted *alr2269*, *alr4893,* and *alr0075* in *Anabaena* 7120, generating the completely segregated *Δalr2269*, *Δalr4893*, and *Δalr0075* mutants (Fig. 1B; Fig. S1A). To confirm this result, we also generated an *alr2269* insertion mutant, *alr2269M*, by inserting a chloramphenicol- and erythromycin-resistance cassette (C.CE2) into the Mlu I site of *alr2269* (Fig. S2A). PCR detection showed a complete segregation of the insertion mutant. To strengthen the PCR examination result, we performed quantitative PCR to evaluate the wild-type copy number in the mutant. As shown in Fig. 1C, the wild-type form accounted for about 2 in 10,000 genome copies (virtually undetectable, the same level as the background), indicating that the *alr2269M* strain was indeed completely segregated. Then, we checked the Alr2269 protein in the mutants by Western blotting. Alr2269 was present in the wild type but not in the *alr2269M* (Fig. 2A) and *Δalr2269* (Fig. 2B) mutants, and complementation of the *alr2269M* strain with *alr2269* on a replicative plasmid restored the band of Alr2269 (Fig. 2A). Alr2269 was also detectable in the *Δalr4893* and *Δalr0075* mutants (Fig. 2B).

Considering that Alr2269 is one of the three Omp85 proteins in *Anabaena* 7120, we investigated whether Alr2269 influences the insertion of β-barrel proteins into the outer membrane (OM). We isolated and purified the OM from the wild type (WT) and *alr2269M* and verified the purity of OM samples through SDS-PAGE analysis (Fig. S3A) and spectral scanning (Fig. S3B). Compared with total membranes, the purified OM showed much less protein bands and lost the characteristic chlorophyll *a* absorption peaks at 440 and 680 nm, retaining the carotenoid absorption peak at 487 nm.

**Fig 3:**
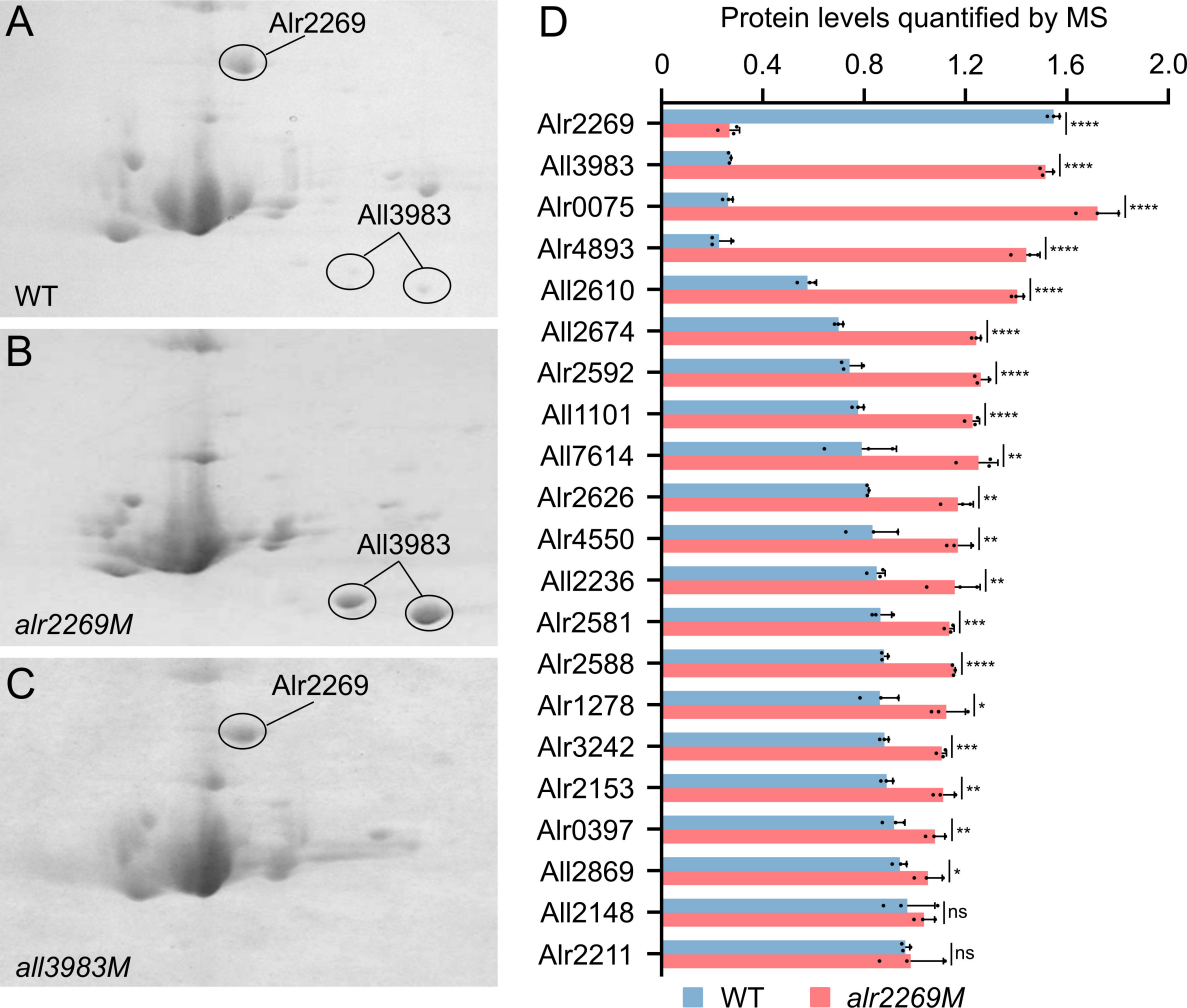
Proteomic analyses of outer membrane proteins in *Anabaena* 7120 (WT) and mutants. (**A–C**) Two-dimensional (2-D) gel electrophoretogram of outer membrane (OM) proteins of the WT, *alr2269M,* and *all3983M*. Protein spots were identified by using tandem mass spectrometry. (**D**) Quantitative analysis of β-barrel proteins and All3983 in the OM of WT and *alr2269M* (see Table S2 for a full list of OM proteins). All values are plotted as mean ± SD (*n* = 3). Asterisks denote significant differences by two-tailed Student’s *t*-test (*P < 0.05, **P < 0.01, ***P < 0.001, ****P < 0.0001). ns, not significant.

We then subjected the OM samples of the WT and *alr2269M* to two-dimensional (2-D) gel electrophoresis. The 2-D patterns were quite similar to each other, except for the disappearance of one spot and the apparent accumulation of two others in *alr2269M* (Fig. 3A and B). Mass spectrometric analysis of these differentially expressed protein spots revealed that the missing protein was Alr2269, as expected for the insertion mutant, while the two accumulated spots corresponded to the same protein, All3983, a predicted S-layer protein. Its N-terminal amino acid no. 21-210 is 31.79% identical to the SLH domain of the surface layer (S-layer) protein SbpA of *Lysinibacillus sphaericus* (36) (Q9RER7) (E-value 2e-17).

To further evaluate the influence of Alr2269 on the OM protein profile, we performed TMT-labeled quantitative proteomic analysis of the OM samples. Among the mass spectrometry-detected proteins, 102 were identified as OM proteins using the functional annotation web server DAVID (37) and the protein subcellular localization prediction tool PSORTb v3.0.3 (38) (union of the two lists, see Table S2). β-Barrel proteins were predicted using the Deep TMHMM (39). The proteomic analysis showed that no β-barrel proteins disappeared in the OM of *alr2269M* compared to the WT; in contrast, most β-barrel proteins were upregulated or non-regulated in the mutant (Table S2, with some of the upregulated proteins shown in Fig. 3D). The proteomic data confirmed the significant upregulation of All3983 and the other two Omp85 proteins Alr4893 and Alr0075 in the *alr2269M* mutant relative to the WT.

### Identification of *all3983* as a Ca^2+^-responsive marker in the *alr2269* mutant

In the absence of Alr2269, the abundance of All3983 significantly increased, suggesting a transcriptional or post-transcriptional regulation of *all3983* in the mutant, so we employed the luciferase genes *luxAB* to monitor the promoter activity of *all3983* in the WT and *alr2269M*. P*_all3983_-luxAB* was integrated into the genome via single homologous recombination, generating strains WT::P*_all3983_-luxAB* and *alr2269M*::P*_all3983_-luxAB*.

**Fig 4:**
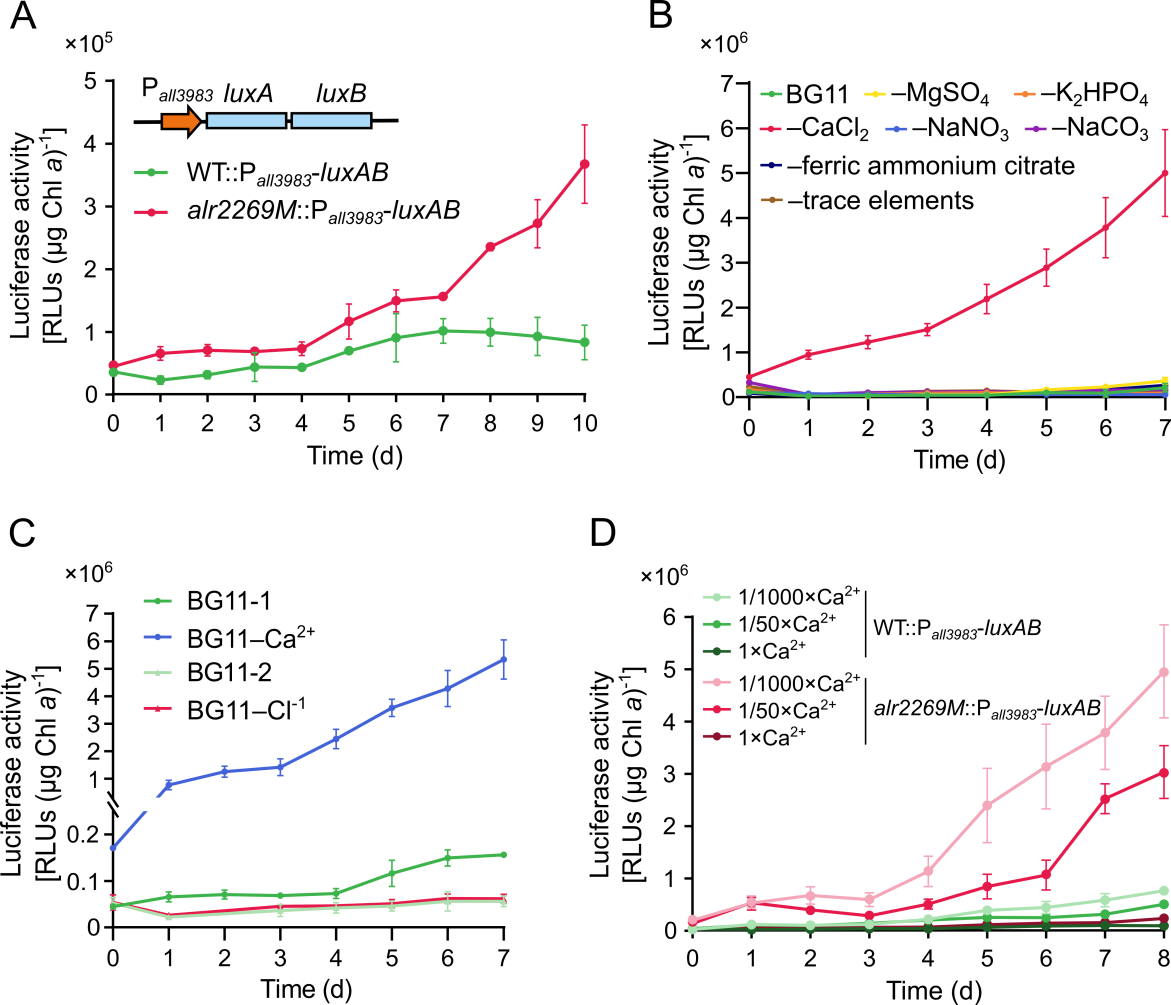
Upregulation of *all3983* in *alr2269M* in response to decrease of (Ca^2+^). (**A**) Expression of *luxAB* from the promoter of *all3983* (P*_all3983_-luxAB*) in the WT or *alr2269M*, as shown with the luciferase activity. The growth of *Anabaena* strains would elevate the pH of the medium, lowering the concentration of free Ca^2+^ (through the reaction with CO_3_^2-^). The inset shows the structure of P*_all3983_-luxAB*. (**B**) Responses of *alr2269M*::P*_all3983_-luxAB* to removal of each component (indicated with the minus sign, for example, -MgSO_4_) from BG11. (**C**) Responses of *alr2269M*::P*_all3983_-luxAB* to removal of Ca^2+^ (CaCl_2_ was replaced with NaCl) and Cl^-^ (CaCl_2_ was replaced with Ca(NO_3_)_2_, and NaCl was removed). BG11-1 serves as the control for BG11-Ca^2+^, while BG11-2 serves as the control for BG11-Cl^-^. (**D**) Responses of WT::P*_all3983_-luxAB* and *alr2269M*::P*_all3983_-luxAB* to [Ca^2+^] in the medium. 1 × Ca^2+^ refers to BG11 (240 µM Ca^2+^); 1/50 × Ca^2+^, BG11 with 4.8 µM Ca^2+^; 1/1000×Ca^2+^, BG11 with 0.24 µM Ca^2+^.

After transfer of cells into the fresh BG11 medium, the transcription of *all3983* gradually increased over time in the *alr2269* mutant but remained nearly constant in the WT (Fig. 4A). The upregulation of *all3983* could be the major reason for the accumulation of All3983 in *alr2269M*. We wondered if this can be enhanced by nutrient deficiency in the medium; therefore, we tested the response of P*_all3983_-luxAB* in the *alr2269* mutant to removal of each component from BG11 (Fig. 4B). Over 7 days, luciferase activity increased markedly only in the medium with CaCl_2_ omitted but remained at baseline levels when other components were removed. To differentiate between Ca^2+^ and Cl^-^, we replaced CaCl_2_ with Ca(NO_3_)_2_ or NaCl. Only lack of Ca^2+^ induced the upregulation of *all3983* (Fig. 4C). Thus, the inducing factor should be lack of Ca^2+^ rather than Cl^-^. Furthermore, when WT::P*_all3983_-luxAB* and *alr2269M*::P*_all3983_-luxAB* were grown in BG11 with the [Ca^2+^] reduced to 1/50 or 1/1,000 of the standard level (240 µM), *alr2269M*::P*_all3983_-luxAB* showed elevated expression of *all3983* relative to the same strain in the standard BG11 medium (Fig. 4D). These findings strongly indicated that Alr2269 and All3983 are related to Ca^2+^ homeostasis.

We also generated an insertion mutant of *all3983* (Fig. S2B), and the mutant *all3983M* was completely segregated as shown with PCR and quantitative PCR (Fig. 1D). 2-D gel electrophoresis of the OM proteins indicated that All3983 was absent while the abundance of Alr2269 remained unchanged in *all3983M* (Fig. 3A and C).

### Role of Alr2269 in Ca^2+^ uptake

Given that *all3983* was upregulated in response to Ca^2+^ depletion in the absence of Alr2269, and that both Alr2269 and All3983 are outer membrane proteins, we hypothesized that Alr2269 and All3983 may contribute to Ca^2+^ utilization. To test this, Ca^2+^ uptake assays were conducted in the WT and mutant strains, and their growth was compared under Ca^2+^-limiting conditions.

**Fig 5:**
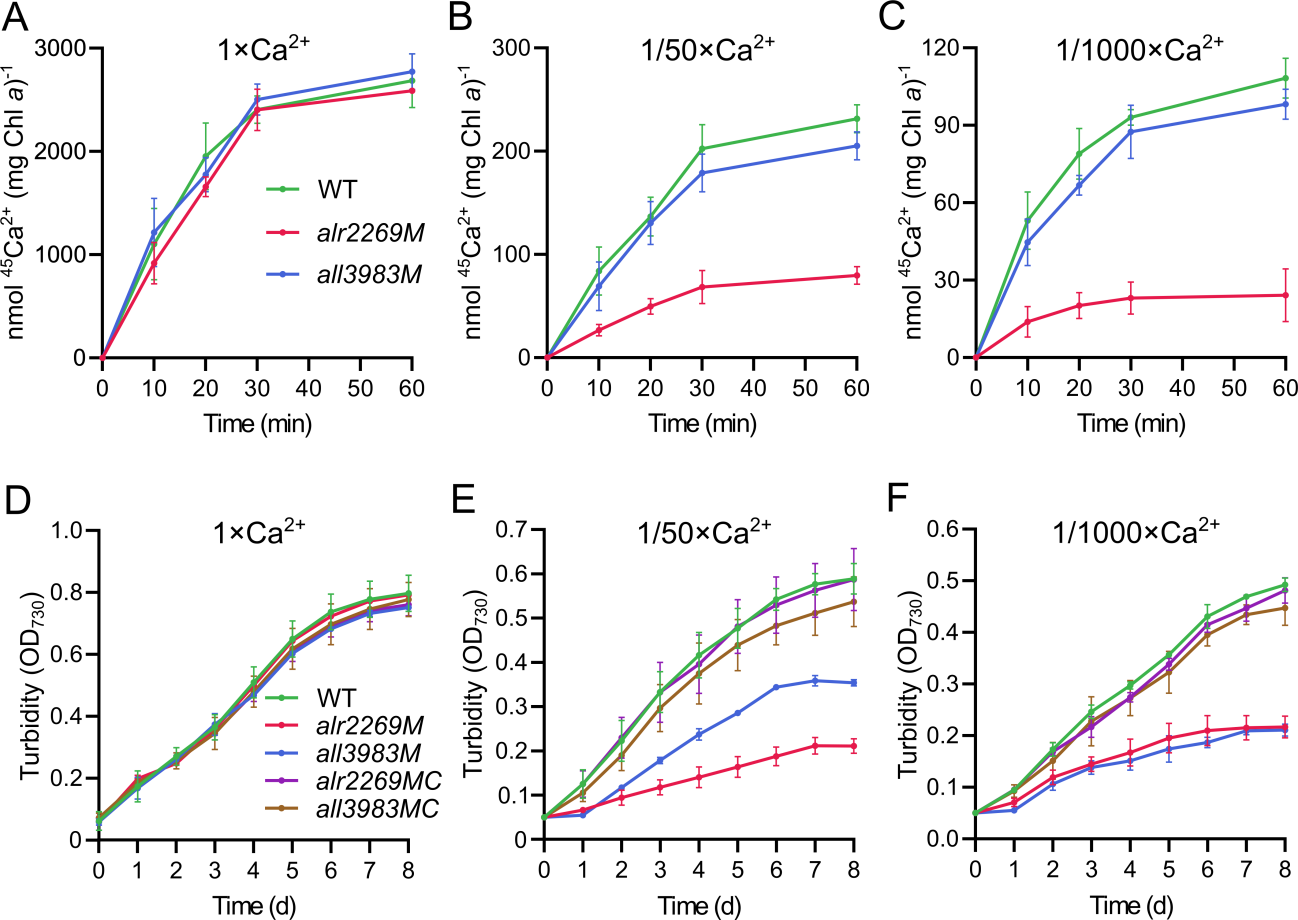
Ca^2+^ uptake and utilization in *Anabaena* 7120 and derivative strains. (**A–C**) Time course of intracellular ^45^Ca^2+^ content in the WT, *alr2269M*, and *all3983M* strains after incubation in 1 × Ca^2+^, 1/50 × Ca^2+^, and 1/1,000 × Ca^2+^ media (^45^Ca^2+^ supplemented as 1/10,000 of total Ca^2+^). (**D–F**) Growth curves of the WT, *alr2269M*, *all3983M*, *alr2269MC* (*alr2269M* complemented with *alr2269*), and *all3983MC* (*all3983M* complemented with *all3983*) in 1 × Ca^2+^, 1/50 × Ca^2+^, and 1/1,000 × Ca^2+^ media.

Ca^2+^ uptake was assayed using the ^45^Ca^2+^ isotope. As shown in Fig. 5A, no significant difference in ^45^Ca^2+^ uptake between strains was observed in standard BG11. However, when the [Ca^2+^] was reduced to 1/50 or 1/1,000 of the standard [Ca^2+^], *alr2269M* displayed a marked decrease in ^45^Ca^2+^ uptake compared with the WT, whereas *all3983M* was only slightly reduced in ^45^Ca^2+^ uptake (Fig. 5B and C). Notably, some Ca^2+^ uptake activities were still detectable in the absence of Alr2269.

The growth of these strains was also compared under Ca^2+^-limiting conditions. In BG11, *alr2269M* and *all3983M* grew as the wild type (Fig. 5D); however, in BG11 with 1/50 standard [Ca^2+^], the two mutants showed significantly reduced growth rates compared with the WT, with the growth retardation of *alr2269M* more pronounced (Fig. 5E); when [Ca^2+^] was reduced to 1/1,000, the two mutants showed comparably significant reduction in growth (Fig. 5F). To exclude the possibility of polar effects or second mutations, we complemented the mutants with the corresponding wild-type genes. The complemented strains *alr2269MC* and *all3983MC* grew as the wild type under the low Ca^2+^ conditions (Fig. 5E and F).

Because Ca^2+^ concentration is tightly controlled in bacterial cells by orchestration of efflux, uptake, and sequestration (such as binding to proteins) of Ca^2+^ (40), the intracellular free Ca^2+^ concentrations ([Ca^2+^]*_i_*) of the wild type and *alr2269M* would not show differences as large as that between Ca^2+^ uptake activities of the two strains. Even so, in Ca^2+^-free or 1/50 × Ca^2+^ medium, [Ca^2+^]*_i_* was significantly lowered in *alr2269M* compared with the wild type and restored to the wild type level in the complemented strain *alr2269MC*; in the 1×Ca^2+^ medium, *alr2269MC* showed even a higher [Ca^2+^]*_i_* than the wild type (Fig. S4).

We also examined the growth of the independent *alr2269* mutant, △*alr2269*, under calcium-limiting conditions and found the same phenotype as *alr2269M* (Fig. S1B). However, the deletion mutants of the other two Omp85 protein genes, *Δalr4893* and *Δalr0075*, grew as the wild type in the low Ca^2+^ medium.

**Fig 6:**
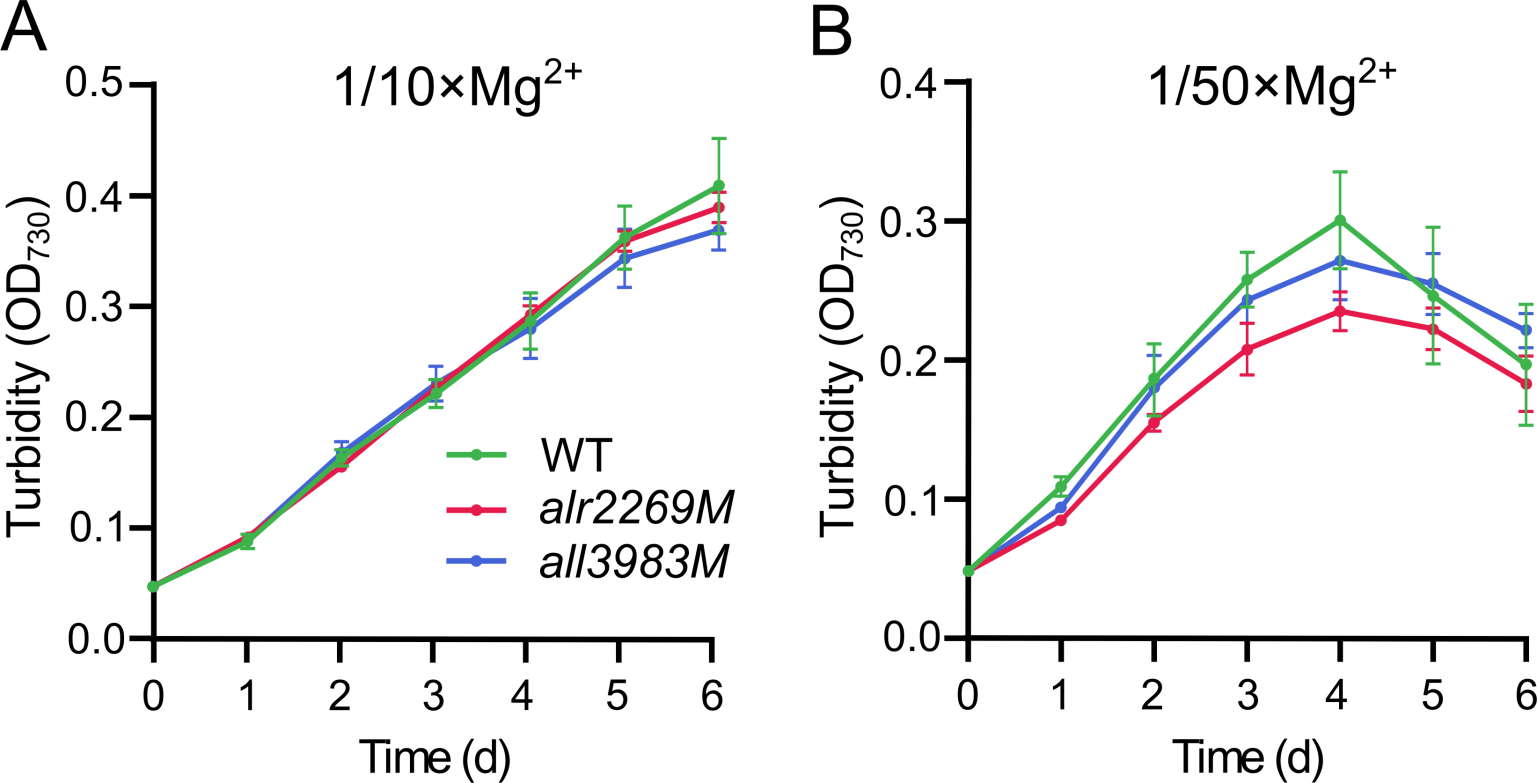
Growth of *Anabaena* 7120 and the *alr2269M*, *all3983M* mutants in BG11 with 1/10 × Mg^2+^ (**A**) or 1/50 × Mg^2+^ (**B**). The BG11 medium contains 300 µM MgSO_4_, while the 1/10 × Mg^2+^ and 1/50 × Mg^2+^ media contain 300 µM SO_4_^2-^ (MgSO_4_ and Na_2_SO_4_) and 30 µM or 6 µM Mg^2+^ (MgSO_4_).

Furthermore, we wondered whether Alr2269 is involved in utilization of Mg^2+^, Mg^2+^, and Ca^2+^ both belong to group IIA of the periodic table, thus are more similar to each other than other divalent cations. In BG11 with 1/10 of standard [Mg^2+^], *alr2269M* and *all3983M* grew as the wild type (Fig. 6A); at 1/50 of standard [Mg^2+^], *alr2269M* showed slightly slower growth than the wild type and *all3983M*, and all these strains started to decline in turbidity after 4 days (Fig. 6B). As the most abundant intracellular divalent cation, Mg^2+^ is involved in the synthesis of chlorophyll and many other biochemical reactions; thus, it was not feasible to further reduce the [Mg^2+^] in the medium. Considering 1/50 [Ca^2+^] (4.8 µM) and 1/50 [Mg^2+^] (6.0 µM) are at comparable levels, the remarkably different effects of Alr2269 on the growth under Ca^2+^- or Mg^2+^-limiting conditions (Fig. 5E and 6B) suggest that Alr2269 is a relatively specific channel for Ca^2+^ uptake.

## DISCUSSION

Toc75 homologs in cyanobacteria, such as Slr1227 in *Synechocystis* and Alr2269 in *Anabaena*, have long been regarded as indispensable for cell viability, given their presumed role in outer membrane biogenesis. Previous studies, in which none of the mutants of these genes was completely segregated, lend support to this notion (29, 31).

In *Synechocystis* sp. PCC 6803, SynToc75 (Slr1227) is the only Omp85 protein. Under our conditions, an *slr1227* mutant of *Synechocystis* was indeed partially segregated as previously reported (29). However, *Anabaena* 7120 possesses three Omp85 proteins, of which at least Alr2269 and Alr4893 are functionally redundant. In this study, we generated two independent *alr2269* mutants of *Anabaena* 7120, and the mutants were both completely segregated. Fourfold evidence, including PCR, qPCR (Fig. 1C), Western blotting (Fig. 2), and 2-D gel electrophoresis (Fig. 3B), confirmed the complete absence of Alr2269 in *alr2269M*. These results clearly indicated that Alr2269 is non-essential for *Anabaena* 7120 in BG11. The discrepancy between our results and previous reports could be due to independent microevolution of *Anabaena* 7120 in laboratories. In our previous studies, over 300 mutations were identified between “PCC 7120” isolates from different laboratories and referred to these isolates as “substrains of PCC 7120”; occasionally, the same genotype may cause different phenotypes in these substrains (41). Perhaps, due to the divergence in genomic sequence, *alr2269* is non-essential in the IHB substrain used in our studies but essential in the other substrains (31, 32).

To evaluate the influence of the inactivation of *alr2269* on the OM protein profile, we conducted 2-D gel electrophoresis and TMT-labeled quantitative proteomic analyses to compare the mutant and the wild type. The 2-D gel electrophoresis showed the absence of Alr2269 and the accumulation of All3983 in the *alr2269* mutant compared with the WT; the quantitative proteomic analysis revealed the upregulation of many more proteins, including All3983 and many β-barrel proteins in the mutant. No OM β-barrel protein disappeared in *alr2269M*. Apparently, Alr2269 is not essential for β-barrel protein integration into the OM. The upregulation of Alr4893, structurally similar to Alr2269, might have partially compensated for the inactivation of *alr2269*.

The accumulation of All3983 is one of the most prominent changes caused by inactivation of *alr2269*. Investigation of the effect of each component in the medium on the expression of *all3983* led us to the finding that calcium deficiency induced the upregulation of *all3983* in the *alr2269* mutant. It is our hypothesis that there is a Ca^2+^-mediated signal transduction pathway between Alr2269 and the transcription of *all3983*. In other words, Alr2269 is probably involved in Ca^2+^ utilization, in particular transport across the OM. The Ca^2+^ uptake assays and the tests of growth in low-Ca^2+^ medium substantiated the role of Alr2269 in Ca^2+^ utilization, and the role of Alr2269 was confirmed by complementation of the *alr2269* mutant. Because Alr2269 is not required for β-barrel protein integration into the OM, this protein itself should be the main channel for import of Ca^2+^ across the OM. The upregulated Alr4893 and many other β-barrel proteins may account for the remaining Ca^2+^ uptake in the *alr2269* mutant (Fig. 5B and C). In the presence of Alr2269, Alr4893 and Alr0075 were not upregulated; therefore, they should have even less influence on the growth in low Ca^2+^ medium (Fig. S1B).

Although Mg^2+^ uptake was not assayed, the remarkable difference between the role of Alr2269 in growth under low-Mg^2+^ and low-Ca^2+^ conditions led us to propose that the Alr2269 channel is relatively specific for the import of Ca^2+^. The β-barrel architecture and conserved POTRA domains of Alr2269 align with features of the chloroplast Toc75 channel (27). These domains likely contribute to the gating mechanism that allows selective Ca^2+^ permeation.

Unlike Alr2269, All3983 is predicted to be an S-layer protein. In general, S-layer proteins are anchored to the cell surface through interactions between SLH domains and specific polysaccharide components in the cell wall, organized as two-dimensional crystalline arrays (42). As shown in Fig. 5B and C, All3983 is basically not involved in Ca^2+^ uptake. However, it may facilitate recruitment of Ca^2+^ to the cell surface and strengthen the stability of the OM under Ca^2+^-limiting conditions, thus is required for the growth in low-Ca^2+^ medium (Fig. 5E and F).

In summary, our results establish Alr2269 (AnaToc75) as the main channel protein across the OM of *Anabaena* for uptake of Ca^2+^ at low concentrations. In BG11 with standard [Ca^2+^], Alr2269 is dispensable (Fig. 5D) because diffusion through other β-barrel channels in the OM could supply sufficient Ca^2+^ to the intracellular space. In most freshwater environments, calcium is not a limiting factor for proliferation of cyanobacteria. However, free Ca^2+^ may be reduced to low concentrations when cyanobacteria grow to relatively high densities and the pH increases (due to the consumption of CO_2_). The evolution of a cyanobacterial Toc75 homolog into a Ca^2+^ channel provides an example of additional functions of Omp85 proteins in bacteria other than protein integration and translocation.

## MATERIALS AND METHODS

### Strains and culture conditions

*Anabaena* 7120 was obtained from the Freshwater Algae Culture Collection at the Institute of Hydrobiology, Chinese Academy of Sciences. *Anabaena* 7120 strains were maintained in BG11 medium (43) at 30°C in the light of 10–20 μE m^−2^ s^−1^ without agitation. When necessary, antibiotics were added to the medium as follows: erythromycin at 5 µg mL^−1^, spectinomycin at 10 µg mL^−1^, and neomycin at 20 µg mL^−1^.

For generation of growth curves at different Ca^2+^ concentrations, *Anabaena* cells were cultured in transparent plastic bottles, with illumination of 30 μE m^−2^ s^−1^ from both upper and lower sides, manually agitated twice a day. Cells washed with Ca^2+^-free BG11 and pre-cultured in BG11 with different Ca^2+^ concentrations were inoculated into the same medium at an initial optical density (OD_730_) of 0.05, and the OD_730_ values were measured daily and plotted against time. For the growth in low Ca^2+^ BG11, illumination from both sides with manual agitation produced more consistent and regular growth curves than illumination only from the upper side with constant agitation on a rotary shaker.

### Construction of plasmids and *Anabaena* strains

Molecular cloning was performed following standard protocols or manufacturer’s instructions. DNA fragments amplified by polymerase chain reactions (PCRs) were confirmed by sequencing after cloning. DNA ligations were conducted either by use of T4 DNA ligase (ligation of compatible dsDNA ends) or 2× MultiF Seamless Assembly Mix (ABclonal, Wuhan, China) (*in vitro* homologous recombination). Plasmids were introduced into the *Anabaena* 7120 strains by conjugation (44). Mutants were generated by deleting a stretch of sequence within a gene using the Cpf1-based genome editing system (35), or by interrupting a gene with an antibiotic resistance cassette using the strategy of *sacB*-based positive selection (45). Complete segregation of mutants was validated by PCR. Construction of plasmids and *Anabaena* strains, and sequences of PCR primers, are described in detail in Table S1.

### Quantitative PCR (qPCR) analyses

Genomic DNA (gDNA) was extracted and purified using a mini-prep method (45). qPCR was performed according to the core principles of the MIQE guidelines (46), using SYBR Green Realtime PCR Master Mix (Toyobo, Osaka, Japan), in 96-well plates on a Bio-Rad CFX96 Real-Time PCR System (Bio-Rad Laboratories, Hercules, USA). Twenty microliters of reaction mixtures contained 10 µL of master mix, 6 pmol of each primer, 10 ng of gDNA, and nuclease-free water. PCRs initiated with 95°C for 3 min, followed by 40 cycles of: 95°C for 15 s, 60°C for 15 s, and 72°C for 15 s. Three no-template controls per plate were included. A pooled gDNA standard (equimolar mix of all samples) was serially diluted twofold for six concentration points and used to generate the standard curve, with each dilution run in triplicate. Amplification efficiency, standard curve linearity R^2^ and single peak melting temperature for each pair of primers are summarized in Table S3. Primers were designed based on unique sequences in the genome, and the specificity of a primer pair was demonstrated by the single band of agarose electrophoresis and the single peak of melting curve. Relative abundance of residual wild-type alleles was normalized with *rnpB* as the reference gene in the chromosomal DNA to correct variations between samples (47), whose efficiency differed by <5% from target genes. Data were analyzed using the 2^−ΔΔ^*^C^*^T^ method (48), and raw C_t_ values and calculated results are listed in Tables S4 and S5.

### Western blot analysis

*Anabaena* 7120 cells were washed with 20 mM Tris-HCl (pH 8.0) containing 1 mM PMSF and resuspended in the same buffer. Cells were broken using a French press (Scientz, Ningbo, China) at a pressure of 15 MPa, followed by centrifugation at 12,000×*g* for 15 min at 4°C. The resulting supernatant was collected as total cell extracts for Western blot analysis. Protein samples were mixed with loading buffer (Zomabio, Beijing, China) and boiled for 5 min. Protein concentrations were determined using the Bradford Protein Assay Kit (CWBio, Taizhou, China). Equal amounts of proteins (20 µg per lane) were resolved by 12% SDS-PAGE and electro-blotted onto nitrocellulose (NC) membranes (MilliporeSigma, Burlington, USA). NC membranes were blocked with 5% skim milk in TBS-1 (25 mM Tris-HCl, 500 mM NaCl, pH 7.5) for 4 h at room temperature, then incubated overnight at 4°C with anti-Alr2269 rabbit antiserum (Qiwei Yicheng, Beijing, China; 1:1000 dilution in 1% skim milk). After washing three times with TBS-1, NC membranes were incubated with HRP-conjugated goat anti-rabbit IgG secondary antibody (Thermo Scientific, Waltham, USA; 1:5000 in 1% skim milk) for 2 h, then washed three times with TBST (25 mM Tris-HCl, 500 mM NaCl, 0.05% Tween-20, pH 7.5). Signals were developed using Omni-ECL Substrate (YamayBio, Shanghai, China) for 1 min and detected with ImageQuant LAS 4000 mini system (GE Healthcare, Chicago, USA).

### Isolation of the outer membrane of *Anabaena* 7120

Outer membrane isolation was performed by aqueous two-phase partitioning followed by sucrose density gradient ultracentrifugation as described (49) with modifications. Briefly, cells from 2 L of culture (OD_730_ ≈ 1.2) were harvested by centrifugation and washed and resuspended with 20 mM potassium phosphate buffer (pH 7.8) and 1% protease inhibitor cocktail (APExBIO, Houston, USA), then stored at −70°C. Frozen cells were disrupted by vortexing with glass beads, the cell suspension was centrifuged at 3,300×*g* for 10 min to pellet debris, and the supernatant was collected. Total membranes were collected by ultracentrifugation (Optima L-100 XP, SW 41 Ti, Beckman Coulter, Brea, USA) at 103,000×*g* for 30 min. The membrane suspension was subjected to aqueous two-phase partitioning using a system containing 6.5% (w/w) dextran T-500, 6.5% (w/w) PEG3350, 0.25 M sucrose, and 5 mM potassium phosphate (pH 7.8). The second partitioning step was performed with a system containing 7.2% (w/w) dextran T-500, 7.2% (w/w) PEG3350, 0.25 M sucrose, and 5 mM potassium phosphate (pH 7.8). The outer membrane was further purified by two rounds of sucrose density gradient ultracentrifugation at 197,000×*g* for 6 h at 4°C. Purity of the isolated outer membrane was analyzed by SDS-PAGE and spectral scanning with UV2450 UV spectrophotometer (Shimadzu, Kyoto, Japan).

### Two-dimensional gel electrophoresis

Total protein samples were dissolved in the solubilization buffer (9 M urea, 4% CHAPS, 2% IPG buffer, 40 mM DTT). An appropriate amount of sample was mixed with the rehydration buffer (8 M urea, 2% CHAPS, 15 mM DTT, 0.5% IPG buffer) to a total volume of 250 µL containing 270 µg proteins and loaded onto immobilized pH gradient (IPG) strips. Isoelectric focusing was performed as follows: 50 V for 12 h (rehydration), 200 V for 1 h, 500 V for 1 h, 1,000 V for 1 h, 2,000 V for 2 h, 3,500 V for 17 h. After isoelectric focusing, IPG strips were equilibrated by shaking for 15 min in 10-mL equilibration buffer I (0.05 M Tris-HCl, pH 8.8, 6 M urea, 30% [w/v] glycerol, 2% [w/v] SDS, 0.002% [w/v] bromophenol blue, 64.8 mM DTT) followed by 15 min in 10 mL equilibration buffer II (0.05 M Tris-HCl, pH 8.8, 6 M urea, 30% [w/v] glycerol, 2% [w/v] SDS, 0.002% [w/v] bromophenol blue, 0.216 M iodoacetamide). Strips were then rinsed in deionized water and applied to 12% SDS-PAGE gel for second dimension electrophoresis at 20 mA for 0.5 h, followed by 35 mA for 4.5 h. Gels were stained with Coomassie Brilliant Blue.

### TMT-labeled quantitative proteomic analysis

Outer membrane samples (with approximately 100 µg proteins) were subjected to ultrasonic treatment on ice in the lysis buffer (8 M urea, 1% protease inhibitor cocktail). After centrifugation at 12,000×*g* at 4°C for 10 min, the protein solutions were collected and treated with 5 mM dithiothreitol at 56°C, followed by alkylation with 11 mM iodoacetamide in the dark at room temperature. The solutions were diluted with 100 mM Tetraethylammonium bromide (TEAB) solution, lowering the urea concentration to below 2 M. Proteins were digested with trypsin at 37°C (1:50 [w/w], overnight; 1:100 [w/w] for 4 h), and the resulting peptides were desalted using a Strata X C18 SPE column (Phenomenex, Torrance, USA) and vacuum-dried, then dissolved in 0.5 M TEAB buffer and processed with the TMT reagent kit (Thermo Fisher Scientific, Waltham, USA) according to manufacturer′s protocol. After labeling, peptides were analyzed on an EASY-nLC 1000 UPLC system at a constant flow rate of 400 nL/min for NSI source detection, followed by tandem mass spectrometry (MS/MS) on a Q Exactive Plus (Thermo Scientific, Waltham, USA) coupled with the UPLC system. MS/MS data were processed using the MaxQuant search engine (v.1.5.2.8) (50). Protein abundance was quantified based on peptide intensity values. Intensity values of each peptide were normalized across the six samples (3 WT + 3 *alr2269M*) to the average value. The peptide quantification values were then normalized to the median value of each sample. Unique peptides for each protein were identified, and protein quantification values were derived from the median of non-zero unique peptide values. The relative quantification ratio (*alr2269M* / WT) was calculated by comparing the average values of each protein between WT and *alr2269M* (three biological repeats). A Student’s *t*-test was performed on the Log_2_ (*alr2269M* / WT) values to analyze statistical significance.

### Ca^2+^ uptake assays

Ca^2+^ uptake assay was conducted as described (51) with modifications. Cyanobacterial cells were washed three times with Ca^2+^-free BG11 to remove extracellular Ca^2+^, re-suspended in Ca^2+^-free BG11, and cultured for 5–7 days. Ca^2+^ uptake was initiated by re-suspending the Ca^2+^-depleted cells in BG11 containing different concentrations of ^45^Ca^2+^. At various incubation times (0, 10, 20, 30, 60 min), 1-mL aliquots were vacuum-filtered through 0.22-µm NC membranes pre-soaked in deionized water and washed three times with TBS-2 buffer (50 mM Tris-HCl, 150 mM NaCl, pH 7.5). The NC membranes with cells were transferred to scintillation vials containing 2 mL high-efficiency mineral oil scintillator and subjected to counting with the MicroBeta^2^ scintillation counter (PerkinElmer, Waltham, USA). A standard curve of scintillation counts per minute (cpm) against ^45^Ca^2+^ concentrations was established by measuring the radioactivity of serially diluted ^45^Ca^2+^ solutions, and ^45^Ca^2+^ concentrations of samples were interpolated from the standard curve. Chlorophyll *a* was extracted with methanol in the dark and determined based on the absorption coefficient of Chl *a* at OD_664_ (52). ^45^Ca^2+^ imported into cells was calculated based on three biological replicates and expressed in nmol ^45^Ca^2+^ (mg Chl *a*)^−1^.

### Measurements of intracellular free Ca^2+^ using Fura-4 AM

Intracellular free calcium concentrations [Ca^2+^]*_i_* of *Anabaena* 7120 and derivative strains were evaluated using Fura-4 AM as described (53) with modifications. Forty microliters of *Anabaena* cells was collected by centrifugation, washed three times with Ca^2+^-free BG11 and allowed to grow in the same medium (to deplete stored Ca^2+^) for 5 days. The cells were then collected and washed three times with buffer A (50 mM Tris-HCl, 100 mM KCl, 1 mM MgCl₂, pH 7.5) and re-suspended in 1 mL of 0.12 M Tris-HCl (pH 8.0) containing 0.2 mM EDTA, shaken at 30°C for 2 min, and supplemented with MgCl₂ to a final concentration of 1 mM. Subsequently, the cells were washed three times with buffer A, re-suspended in 1 mL of buffer A, and loaded with 2.5 µM Fura-4 AM (Beyotime, Shanghai, China) in the dark at 30°C for 1 h. Again, the cells were washed three times and suspended in 1 mL of buffer A. Then, 500 µL of Fura-4 AM-loaded cells was added to 20 mL of Ca^2+^-free BG11, or 1 × Ca^2+^ BG11, or 1/50 × Ca^2+^ BG11 and incubated for 1 h, collected and re-suspended in 500 µL of buffer A. Fluorescence was recorded using a SpectraMax i3x microplate reader (Molecular Devices, Sunnyvale, USA) with excitation at 488 nm and emission at 520 nm. After permeabilization of cells with 25 µM gramicidin (MedChemExpress, Monmouth Junction, USA), *F*_max_ and *F*_min_ were determined by adding 1 mM CaCl₂ and 10 mM EGTA, respectively. [Ca^2+^]*_i_* was calculated using the formula: [Ca^2+^]*_i_* = *K_d_* × (*F − F*_min_) / (*F*_max_ − *F*). One-way ANOVA analysis followed by LSD post hoc tests was performed to determine significant differences in [Ca^2+^]*_i_* between groups.

### Determination of bacterial luciferase activity

Luciferase activity was measured as described previously (54). Briefly, 1 mL of cells was mixed with 100 µL of 0.1% (v/v) *n*-decanol with 20 mg mL^−1^ BSA. The mixture was placed in an ATP photometer (Berthold, Gosheim, Germany) to measure relative light units (RLUs) for 60 s. In parallel, 1.2 mL of the cell suspension was centrifuged at 12,000×*g* for 5 min, and the pellet was re-suspended in 1.2 mL methanol for extraction of chlorophyll *a*. Luciferase activity was expressed as RLUs (μg Chl *a*)^−1^.

### Bioinformatic analyses

Identification of outer membrane proteins was assisted by using the functional annotation web server DAVID (37) and the protein subcellular localization prediction tool PSORTb v3.0.3 (38). Structures of outer membrane proteins were downloaded from the AlphaFold Protein Structure Database (https://alphafold.ebi.ac.uk/) and visualized using PyMOL software (Version 1.7, Schrödinger, LLC.; https://pymol.org). β-Barrel structures were also predicted using the Deep TMHMM (39).

## SUPPLEMENTAL MATERIAL

Figures S1 to S4 and Tables S1 to S5 jb.00291-25-s0001.docx Fig. S1: Δ*alr2269*, Δ*alr4893*, and Δ*alr0075* mutants of *Anabaena* 7120. Fig. S2: Schematic diagram showing the gene interruption in *alr2269M* and *all3983M* mutants and the strategies for complementation. Fig. S3: Analyses of the purity of outer membrane samples by SDS-PAGE and spectral scanning. Fig. S4: Intracellular Ca^2+^ concentrations ([Ca^2+^]*_i_*) of *Anabaena* 7120 (WT), *alr2269M*, *all3983M*, *alr2269MC*, and *all3983MC*. Table S1: List of strains, plasmids, and primers. Table S2: Comparative proteomic analysis of outer membrane proteins in the wild type (WT) and the *alr2269M* mutant of *Anabaena* sp. PCC 7120. Table S3: Parameters of quantitative polymerase chain reactions (qPCR). Table S4: Threshold cycle (Ct) values for qPCR analysis of *alr2269* and calculations of 2^-△△Ct^. Table S5: Threshold cycle values for qPCR analysis of *all3983* and calculations of 2^-△△Ct^.

ASM does not own the copyrights to Supplemental Material that may be linked to, or accessed through, an article. The authors have granted ASM a non-exclusive, world-wide license to publish the Supplemental Material files. Please contact the corresponding author directly for reuse.

## References

[1] Berridge MJ, Bootman MD, Roderick HL. 2003. Calcium signalling: dynamics, homeostasis and remodelling. Nat Rev Mol Cell Biol 4:517–529. doi:10.1038/nrm1155 12838335

[2] Newton AC, Bootman MD, Scott JD. 2016. Second messengers. Cold Spring Harb Perspect Biol 8:a005926. doi:10.1101/cshperspect.a005926 27481708 PMC4968160

[3] Ubarretxena-Belandia I, Boots JW, Verheij HM, Dekker N. 1998. Role of the cofactor calcium in the activation of outer membrane phospholipase A. Biochemistry 37:16011–16018. doi:10.1021/bi9814181 9843408

[4] Ahvazi B, Boeshans KM, Idler W, Baxa U, Steinert PM. 2003. Roles of calcium ions in the activation and activity of the transglutaminase 3 enzyme. J Biol Chem 278:23834–23841. doi:10.1074/jbc.M301162200 12679341

[5] Karkare S, Yousafzai F, Mitchenall LA, Maxwell A. 2012. The role of Ca^2+^ in the activity of Mycobacterium tuberculosis DNA gyrase. Nucleic Acids Res 40:9774–9787. doi:10.1093/nar/gks704 22844097 PMC3479174

[6] Najafpour MM, Govindjee. 2011. Oxygen evolving complex in photosystem II: better than excellent. Dalton Trans 40:9076. doi:10.1039/c1dt10746a 21735020

[7] Walter J, Selim KA, Leganés F, Fernández-Piñas F, Vothknecht UC, Forchhammer K, Aro EM, Gollan PJ. 2019. A novel Ca^2+^-binding protein influences photosynthetic electron transport in Anabaena sp. PCC 7120. Biochim Biophys Acta 1860:519–532. doi:10.1016/j.bbabio.2019.04.007 31034800

[8] Pitta TP, Sherwood EE, Kobel AM, Berg HC. 1997. Calcium is required for swimming by the nonflagellated cyanobacterium Synechococcus strain WH8113. J Bacteriol 179:2524–2528. doi:10.1128/jb.179.8.2524-2528.1997 9098048 PMC178999

[9] Moon YJ, Park YM, Chung YH, Choi JS. 2004. Calcium is involved in photomovement of cyanobacterium Synechocystis sp. PCC 6803. Photochem Photobiol 79:114–119. doi:10.1111/j.1751-1097.2004.tb09865.x 14974723

[10] Torrecilla I, Leganés F, Bonilla I, Fernández-Piñas F. 2004. A calcium signal is involved in heterocyst differentiation in the cyanobacterium Anabaena sp. PCC7120. Microbiology (Reading, Engl) 150:3731–3739. doi:10.1099/mic.0.27403-0 15528659

[11] Giraldez-Ruiz N, Bonilla I, Fernandez-Piñas F. 1999. Role of external calcium in homeostasis of intracellular pH in the cyanobacterium Anabaena sp. strain PCC7120 exposed to low pH. New Phytol 141:225–230. doi:10.1046/j.1469-8137.1999.00347.x 33862921

[12] Tiwari A, Singh P, Riyazat Khadim S, Singh AK, Singh U, Singh P, Asthana RK. 2019. Role of Ca^2+^ as protectant under heat stress by regulation of photosynthesis and membrane saturation in Anabaena PCC 7120. Protoplasma 256:681–691. doi:10.1007/s00709-018-1328-8 30456698

[13] Cohen SE, Golden SS. 2015. Circadian rhythms in cyanobacteria. Microbiol Mol Biol Rev 79:373–385. doi:10.1128/MMBR.00036-15 26335718 PMC4557074

[14] Pérez-Llorca M, Müller M. 2024. Unlocking nature's rhythms: insights into secondary metabolite modulation by the circadian clock. Int J Mol Sci 25:7308. doi:10.3390/ijms25137308 39000414 PMC11241833

[15] Smith RJ. 1995. Calcium and bacteria. Adv Microb Physiol 37:83–133. doi:10.1016/s0065-2911(08)60144-7 8540424

[16] Torrecilla I, Leganés F, Bonilla I, Fernández-Piñas F. 2000. Use of recombinant aequorin to study calcium homeostasis and monitor calcium transients in response to heat and cold shock in cyanobacteria. Plant Physiol 123:161–176. doi:10.1104/pp.123.1.161 10806234 PMC58991

[17] Barrán-Berdón AL, Rodea-Palomares I, Leganés F, Fernández-Piñas F. 2011. Free Ca^2+^ as an early intracellular biomarker of exposure of cyanobacteria to environmental pollution. Anal Bioanal Chem 400:1015–1029. doi:10.1007/s00216-010-4209-3 20886207

[18] Gangola P, Rosen BP. 1987. Maintenance of intracellular calcium in Escherichia coli. J Biol Chem 262:12570–12574. doi:10.1016/S0021-9258(18)45243-X 2442165

[19] Herbaud ML, Guiseppi A, Denizot F, Haiech J, Kilhoffer MC. 1998. Calcium signalling in Bacillus subtilis. Biochim Biophys Acta 1448:212–226. doi:10.1016/s0167-4889(98)00145-1 9920412

[20] Rosen BP. 1987. Bacterial calcium transport. Biochim Biophys Acta 906:101–110. doi:10.1016/0304-4157(87)90007-4 2436666

[21] Reusch RN, Huang R, Bramble LL. 1995. Poly-3-hydroxybutyrate/polyphosphate complexes form voltage-activated Ca^2+^ channels in the plasma membranes of Escherichia coli. Biophys J 69:754–766. doi:10.1016/S0006-3495(95)79958-1 8519976 PMC1236305

[22] Chang Y, Bruni R, Kloss B, Assur Z, Kloppmann E, Rost B, Hendrickson WA, Liu Q. 2014. Structural basis for a pH-sensitive calcium leak across membranes. Science 344:1131–1135. doi:10.1126/science.1252043 24904158 PMC4119810

[23] Gupta HK, Shrivastava S, Sharma R. 2017. A novel calcium uptake transporter of uncharacterized P-Type ATPase family supplies calcium for cell surface integrity in Mycobacterium smegmatis. mBio 8:e01388-17. doi:10.1128/mBio.01388-17 28951477 PMC5615198

[24] Berkelman T, Garret-Engele P, Hoffman NE. 1994. The pacL gene of Synechococcus sp. strain PCC 7942 encodes a Ca^2+^-transporting ATPase. J Bacteriol 176:4430–4436. doi:10.1128/jb.176.14.4430-4436.1994 8021228 PMC205657

[25] Waditee R, Hossain GS, Tanaka Y, Nakamura T, Shikata M, Takano J, Takabe T, Takabe T. 2004. Isolation and functional characterization of Ca^2+^/H^+^ antiporters from cyanobacteria. J Biol Chem 279:4330–4338. doi:10.1074/jbc.M310282200 14559898

[26] Boradia V, Frando A, Grundner C. 2022. The Mycobacterium tuberculosis PE15/PPE20 complex transports calcium across the outer membrane. PLoS Biol 20:e3001906. doi:10.1371/journal.pbio.3001906 36441815 PMC9731449

[27] Ertel F, Mirus O, Bredemeier R, Moslavac S, Becker T, Schleiff E. 2005. The evolutionarily related beta-barrel polypeptide transporters from Pisum sativum and Nostoc PCC7120 contain two distinct functional domains. J Biol Chem 280:28281–28289. doi:10.1074/jbc.M503035200 15951438

[28] Bölter B, Soll J, Schulz A, Hinnah S, Wagner R. 1998. Origin of a chloroplast protein importer. Proc Natl Acad Sci USA 95:15831–15836. doi:10.1073/pnas.95.26.15831 9861056 PMC28130

[29] Reumann S, Davila-Aponte J, Keegstra K. 1999. The evolutionary origin of the protein-translocating channel of chloroplastic envelope membranes: identification of a cyanobacterial homolog. Proc Natl Acad Sci USA 96:784–789. doi:10.1073/pnas.96.2.784 9892711 PMC15214

[30] Doyle MT, Bernstein HD. 2022. Function of the Omp85 superfamily of outer membrane protein assembly factors and polypeptide transporters. Annu Rev Microbiol 76:259–279. doi:10.1146/annurev-micro-033021-023719 35650668

[31] Nicolaisen K, Mariscal V, Bredemeier R, Pernil R, Moslavac S, López-Igual R, Maldener I, Herrero A, Schleiff E, Flores E. 2009. The outer membrane of a heterocyst-forming cyanobacterium is a permeability barrier for uptake of metabolites that are exchanged between cells. Mol Microbiol 74:58–70. doi:10.1111/j.1365-2958.2009.06850.x 19703111

[32] Schätzle H, Brouwer E-M, Liebhart E, Stevanovic M, Schleiff E. 2021. Comparative phenotypic analysis of Anabaena sp. PCC 7120 mutants of porin-like genes. J Microbiol Biotechnol 31:645–658. doi:10.4014/jmb.2103.03009 33879642 PMC9705863

[33] Koenig P, Mirus O, Haarmann R, Sommer MS, Sinning I, Schleiff E, Tews I. 2010. Conserved properties of polypeptide transport-associated (POTRA) domains derived from cyanobacterial Omp85. J Biol Chem 285:18016–18024. doi:10.1074/jbc.M110.112649 20348103 PMC2878563

[34] Ungerer J, Pakrasi HB. 2016. Cpf1 is a versatile tool for CRISPR genome editing across diverse species of cyanobacteria. Sci Rep 6:39681. doi:10.1038/srep39681 28000776 PMC5175191

[35] Niu TC, Lin GM, Xie LR, Wang ZQ, Xing WY, Zhang JY, Zhang CC. 2019. Expanding the potential of CRISPR-Cpf1-based genome editing technology in the cyanobacterium Anabaena PCC 7120. ACS Synth Biol 8:170–180. doi:10.1021/acssynbio.8b00437 30525474

[36] Ilk N, Völlenkle C, Egelseer EM, Breitwieser A, Sleytr UB, Sára M. 2002. Molecular characterization of the S-layer gene, sbpA, of Bacillus sphaericus CCM 2177 and production of a functional S-layer fusion protein with the ability to recrystallize in a defined orientation while presenting the fused allergen. Appl Environ Microbiol 68:3251–3260. doi:10.1128/AEM.68.7.3251-3260.2002 12089001 PMC126809

[37] Sherman BT, Hao M, Qiu J, Jiao X, Baseler MW, Lane HC, Imamichi T, Chang W. 2022. DAVID: a web server for functional enrichment analysis and functional annotation of gene lists (2021 update). Nucleic Acids Res 50:W216–W221. doi:10.1093/nar/gkac194 35325185 PMC9252805

[38] Gardy JL. 2003. PSORT-B: improving protein subcellular localization prediction for Gram-negative bacteria. Nucleic Acids Res 31:3613–3617. doi:10.1093/nar/gkg602 12824378 PMC169008

[39] Hallgren J, Tsirigos KD, Pedersen MD, Almagro Armenteros JJ, Marcatili P, Nielsen H, Krogh A, Winther O. 2022. DeepTMHMM predicts alpha and beta transmembrane proteins using deep neural networks. bioRxiv. doi:10.1101/2022.04.08.487609

[40] Domínguez DC, Guragain M, Patrauchan M. 2015. Calcium binding proteins and calcium signaling in prokaryotes. Cell Calcium 57:151–165. doi:10.1016/j.ceca.2014.12.006 25555683

[41] Wang Y, Gao Y, Li C, Gao H, Zhang CC, Xu X. 2018. Three substrains of the cyanobacterium Anabaena sp. strain PCC 7120 display divergence in genomic sequences and hetC function. J Bacteriol 200:e00076-18. doi:10.1128/JB.00076-18 29686139 PMC5996696

[42] Zhu C, Guo G, Ma Q, Zhang F, Ma F, Liu J, Xiao D, Yang X, Sun M. 2017. Diversity in S-layers. Prog Biophys Mol Biol 123:1–15. doi:10.1016/j.pbiomolbio.2016.08.002 27498171

[43] Castenholz RW. 2001. Oxygenic photosynthetic bacteria, p 474–487. In Boone DR, Castenholz RW (ed), Bergey’s manual of systematic bacteriology, 2nd ed. Vol. 1. Springer, New York, NY.

[44] Elhai J, Wolk CP. 1988. Conjugal transfer of DNA to cyanobacteria. Methods Enzymol 167:747–754. doi:10.1016/0076-6879(88)67086-8 3148842

[45] Cai YP, Wolk CP. 1990. Use of a conditionally lethal gene in Anabaena sp. strain PCC 7120 to select for double recombinants and to entrap insertion sequences. J Bacteriol 172:3138–3145. doi:10.1128/jb.172.6.3138-3145.1990 2160938 PMC209118

[46] Bustin SA, Benes V, Garson JA, Hellemans J, Huggett J, Kubista M, Mueller R, Nolan T, Pfaffl MW, Shipley GL, Vandesompele J, Wittwer CT. 2009. The MIQE guidelines: minimum information for publication of quantitative real-time PCR experiments. Clin Chem 55:611–622. doi:10.1373/clinchem.2008.112797 19246619

[47] Hou S, Zhou F, Peng S, Gao H, Xu X. 2015. The HetR-binding site that activates expression of patA in vegetative cells is required for normal heterocyst patterning in Anabaena sp. PCC 7120. Sci Bull Sci Found Philipp 60:192–201. doi:10.1007/s11434-014-0724-5

[48] Livak KJ, Schmittgen TD. 2001. Analysis of relative gene expression data using real-time quantitative PCR and the 2^−ΔΔ^^C^^_T_^ Method. Methods 25:402–408. doi:10.1006/meth.2001.1262 11846609

[49] Dong Y, Xu X. 2009. Outer membrane proteins induced by iron deficiency in Anabaena sp. PCC 7120. Prog Nat Sci 19:1477–1483. doi:10.1016/j.pnsc.2009.02.009

[50] Tyanova S, Temu T, Cox J. 2016. The MaxQuant computational platform for mass spectrometry-based shotgun proteomics. Nat Protoc 11:2301–2319. doi:10.1038/nprot.2016.136 27809316

[51] Pandey PK, Singh BB, Mishra R, Bisen PS. 1996. Ca^2+^ uptake and its regulation in the cyanobacterium Nostoc MAC. Curr Microbiol 32:332–335. doi:10.1007/s002849900059 8661678

[52] Mackinney G. 1941. Absorption of light by chlorophyll solutions. J Biol Chem 140:315–322. doi:10.1016/S0021-9258(18)51320-X

[53] Gangola P, Rosen BP. 1987. Maintenance of intracellular calcium in Escherichia coli. J Biol Chem 262:12570–12574. doi:10.1016/S0021-9258(18)45243-X 2442165

[54] Wolk CP, Cai Y, Panoff JM. 1991. Use of a transposon with luciferase as a reporter to identify environmentally responsive genes in a cyanobacterium. Proc Natl Acad Sci USA 88:5355–5359. doi:10.1073/pnas.88.12.5355 11607193 PMC51871

