# Supplementary material for: AnaToc75 (Alr2269) mediates calcium uptake across the outer membrane in Anabaena sp. PCC 7120
Source: J Bacteriol. 2025 Sep 18;207(10):e00291-25. doi: 10.1128/jb.00291-25 (PMC12548389; doi:10.1128/jb.00291-25)
Supplement: Figures S1 to S4 and Tables S1 to S5 — Fig. S1: Δalr2269, Δalr4893, and Δalr0075 mutants of Anabaena 7120. Fig. S2: Schematic diagram showing the gene interruption in alr2269M and all3983M mutants and the strategies for complementation. Fig. S3: Analyses of the purity of outer membrane samples by SDS-PAGE and spectral scanning. Fig. S4: Intracellular Ca2+ concentrations ([Ca2+]i) of Anabaena 7120 (WT), alr2269M, all3983M, alr2269MC, and all3983MC. Table S1: List of strains, plasmids, and primers. Table S2: Comparative proteomic analysis of outer membrane proteins in the wild type (WT) and the alr2269M mutant of Anabaena sp. PCC 7120. Table S3: Parameters of quantitative polymerase chain reactions (qPCR). Table S4: Threshold cycle (Ct) values for qPCR analysis of alr2269 and calculations of 2-△△Ct. Table S5: Threshold cycle values for qPCR analysis of all3983 and calculations of 2-△△Ct. [file jb.00291-25-s0001.docx]

**Supporting information**

**Fig. S1** **Δ*alr2269*, Δ*alr4893* and Δ*alr0075* mutants of *Anabaena* 7120.** (A) Deletions (black box) in the genome of Δ*alr2269*, Δ*alr4893* and Δ*alr0075* mutants. Primers for genotyping the mutants are indicated. (B) Growth of *Anabaena* 7120 (WT) and the three mutants in 1/1000×Ca^2+^ medium.

**Fig. S2 Schematic diagram showing the gene interruption in *alr2269M* and *all3983M* mutants and the strategies for complementation.** (**A**) Interruption of *alr2269* with the C.CE2 cassette (Cm^r^ and Em^r^) and complementation of the mutant with the replicative plasmid carrying *alr2269*. -1973 to 2545 bp, the 5′ to 3′ ends of the DNA fragment relative to the start codon of *alr2269*. alr2269-1 and alr2269-2, primers for PCR examination of the insertion mutant; alr2269-q-F and alr2269-q-R, primers for qPCR. (**B**) Interruption of *all3983* with the C.CE2 cassette and complementation of the mutant with *all3983* on a plasmid integrated into the genome via single-crossover recombination. -761 to 1411 bp, the 5′ to 3′ ends of the DNA fragment relative to the start codon of *all3983*. all3983-3 and all3983-4, primers for PCR examination of the mutant; all3983-q-F and all3983-q-R, primers for qPCR.

**Fig. S3 Analyses of the purity of outer membrane samples by SDS-PAGE and spectral scanning.** (**A**) SDS-PAGE analysis of total membranes (lanes 1 and 2) and the isolated outer membrane (lanes 3 and 4) of *Anabaena* 7120 (1 and 3) and the *alr2269M* mutant (2 and 4). The SDS-PAGE gel was stained by Coomassie Brilliant Blue. (**B**) Spectral scanning (300-800 nm) of total membrane (TM) and outer membrane (OM) samples. WT, wild type.

**Fig. S4 Intracellular Ca^2+^ concentrations ([Ca^2+^]*_i_*) of *Anabaena* 7120 (WT), *alr2269M*, *all3983M*, *alr2269MC* and *all3983MC*.** Cells grown in Ca^2+^-free medium for 5 d were loaded with Fura-4 AM, then transferred to the indicated medium for 1 h. (A) Transfer to Ca^2+^-free or 1×Ca^2+^ medium for 1 h. (B) Transfer to Ca^2+^-free or 1/50×Ca^2+^ medium for 1 h. Data are means ± SD of 3 biological replicates. Statistical significance is indicated as follows: *, *p* < 0.05; **, *p* < 0.01; ***, *p* < 0.001.

**Table S1 List of strains, plasmids and primers**

| **Strains** | **Derivation and relevant characteristics *^a^*** | | **Reference or source** |
| --- | --- | --- | --- |
| WT | Wild type of *Anabaena* sp. PCC 7120 *^b^* | | FACHB *^c^* |
| *alr2269M* | Cm^r^Em^r^, *alr2269* mutant, with the C.CE2 cassette (Cm^r^/Em^r^) inserted into the MluI site of *alr2269*, generated by homologous double-crossover recombination between pHB2095 and the chromosomal DNA of the WT | | This study |
| *all3983M* | Cm^r^Em^r^, *all3983* mutant, with C.CE2 inserted into the HpaI site of *all3983*, generated by homologous double-crossover recombination between pHB2103 and the chromosomal DNA of the WT | |  |
| *alr2269MC* | Cm^r^ Em^r^Nm^r^, a complementation strain of *alr2269M*, generated by introduction of pHB2925 bearing *alr2269* into *alr2269M* | |  |
| *all3983MC* | Cm^r^Em^r^Nm^r^, a complementation strain of *all3983M*, generated by homologous single-crossover recombination between pHB5712 bearing *all3983* and the region upstream of the insertion site within *all3983* in *all3983M* | |  |
| Δ*alr2269* | *alr2269* mutant, with *Anabaena* chromosomal bp 2731307-2733808 deleted, generated by introducing the Cpf1-based genome-editing plasmid pHB7861 into the WT, followed by removal of the plasmid | |  |
| Δ*alr4893* | *alr4893* mutant, with *Anabaena* chromosomal bp 5831763-5833793 deleted, generated by introducing the Cpf1-based genome-editing plasmid pHB8245 into the WT, followed by removal of the plasmid | |  |
| Δ*alr0075* | *alr0075* mutant, with *Anabaena* chromosomal bp 79339-80814 deleted, generated by introducing the Cpf1-based genome-editing plasmid pHB8272 into the WT, followed by removal of the plasmid | |  |
| WT::P*_all3983_*-*luxAB* | Sm^r^Sp^r^, the strain monitoring the transcription of *all3983* with *luxAB* in the WT background, generated by homologous single-crossover recombination between pHB2462 bearing P*_all3983_-luxAB-*Ω (Sm^r^/Sp^r^ cassette) and the chromosomal DNA of the WT | |  |
| *alr2269M*::P*_all3983_*-*luxAB* | Sm^r^ Sp^r^, the strain monitoring the transcription of *all3983* with *luxAB* in the *alr2269*-null background, generated by homologous single-crossover recombination between pHB2462 and the chromosomal DNA of *alr2269M* | |  |
|  | | | |
| **Plasmids** | **Descriptions** | | **Reference or source** |
| pMD18-T | Ap^r^, a T-vector | | Takara |
| pRL598 | Cm^r^Em^r^Km^r^, cloning vector with the C.CE2 cassette | | Black and Wolk, 1994 |
| pRL277 | Sm^r^Sp^r^, *sacB*-bearing cloning vector | | Black et al., 1993 |
| pDC8 | Km^r^ (Nm^r^)Cm^r^, a shuttle vector | | Xu et al., 1993 |
| pRL58 | Ap^r^Sm^r^Sp^r^, *luxAB*-Ω cassette | | Black et al., 1993 |
| pCpf1b-Km | Km^r^ (Nm^r^), CRISPR-pCpf1b genome editing vector | | Niu et al., 2018 |
| pCpf1b-Sp | Sm^r^Sp^r^, CRISPR-pCpf1b genome editing vector | |  |
| pHB2062 | Ap^r^, PCR fragment (*Anabaena* chromosomal bp 2731523-2733673) containing most of the encoding region of *alr2269* (217 bp to 2367 bp), amplified with primers alr2269-1/alr2269-2, cloned into pMD18-T | | This study |
| pHB2064 | Ap^r^Cm^r^Em^r^, C.CE2 excised from pRL598 with XbaI, blunted with T4 DNA polymerase, cloned into MluI-cut and T4 DNA polymerase-blunted pHB2062 | |  |
| pHB2095 | Cm^r^Sp^r^Em^r^, the DNA fragment with C.CE2 inserted into *alr2269*, excised from pHB2064 with KpnI and HindIII, blunted with T4 DNA polymerase, cloned into XbaI-digested and T4 DNA polymerase-blunted pRL277 | |  |
| pHB2097 | Ap^r^, PCR fragment (*Anabaena* sp. chromosomal bp 4799083-4796976) containing *all3983*, amplified with primers all3983-1/all3983-2, cloned into pMD18-T | |  |
| pHB2098 | Ap^r^Cm^r^Em^r^, C.CE2 excised with XbaI from pRL598, blunted with T4 DNA polymerase, ligated with HpaI-cut pHB2097 | |  |
| pHB2103 | Cm^r^Em^r^Sp^r^, the DNA fragment with C.CE2 inserted into *all3983*, excised from pHB2098 with KpnI and PstI, blunted with T4 DNA polymerase, cloned into XbaI-digested and T4 DNA polymerase-blunted pRL277 | |  |
| pHB2892 | Ap^r^, PCR fragment (*Anabaena* sp. chromosomal bp 2729334-2733851) containing *alr2269*, amplified with primers alr2269C-1/alr2269C-2, cloned into pMD18-T | |  |
| pHB2925 | Km^r^, the DNA fragment containing *alr2269*, excised from pHB2892 with BamHI and SalI, cloned into BamHI/SalI-cut pDC8 | |  |
| pHB2400 | Ap^r^Sp^r^, *luxAB*-Ω excised from pRL58 with SmaI, cloned into HpaI-cut pHB2097, generating P*_all3983_*-*luxAB*-Ω | |  |
| pHB2462 | Km^r^Sp^r^, P*_all3983_*-*luxAB*-Ω excised from pHB2400 with DraI, cloned into XhoI-cut and T4 DNA pol.-blunted pRL278 | |  |
| pHB5712 | Em^r^Sp^r^, *all3983* (-716 bp to 1411 bp) excised from pHB2097 with XbaI and PstI, cloned into XbaI/PstI-cut pRL277 | |  |
| pHB7837 | Sp^r^, the short DNA fragment generated by annealing oligos gRNA-2269-F and gRNA-2269-R, cloned into AarI-cut pCpf1b-sp | |  |
| pHB7861 | Sp^r^, the DNA fragment overlapping the 5′ end of *alr2269* (*Anabaena* chromosomal bp 2730707-2731306), generated by PCR using primers Cr-2269-F1/Cr-2269-R1, and the DNA fragment downstream of *alr2269* (*Anabaena* chromosomal bp 2733809-2734435), generated by PCR using primers Cr-2269-F2/Cr-2269-R2, cloned into the BamHI/BglII-cut pHB7837 through seamless assembly (in vitro recombination) | |  |
| pHB8240 | Km^r^, the DNA fragment overlapping the 5′ end of *alr4893* (*Anabaena* chromosomal bp 5831607-5832630), generated by PCR using primers Cr-4893-F1/Cr-4893-R1, and the DNA fragment overlapping the 3′ end of the *alr4893* (*Anabaena* chromosomal bp 5833746-5834744) with primers Cr-4893-F2/Cr-4893-R2, cloned into the BamHI/BglII-cut pCpf1b-Sp through seamless assembly | |  |
| pHB8245 | Km^r^, the short DNA fragment generated by annealing oligos gRNA-4893-F/gRNA-4893-R, cloned into the AarI-cut pHB8240 | |  |
| pHB8271 | Sp^r^, the DNA fragment overlapping the 5′ end of *alr0075* (*Anabaena* chromosomal bp 78302-79344), generated by PCR using primers Cr-0075-F1/Cr-0075-R1, and DNA fragment overlapping the 3′ end of *alr0075* (*Anabaena* chromosomal bp 79862-80836), generated by PCR using primers Cr-0075-F2/ Cr-0075-R2, cloned into the BamHI/BglII-cut pCpf1b-Km through seamless assembly | |  |
| pHB8272 | Sp^r^, the short DNA fragment generated by annealing oligos gRNA-0075-F/gRNA-0075-R, cloned into the AarI-cut pHB8271 | |  |
|  | | | |
| **Genes** | **Primers** | \| **Sequences（5′ → 3′）** \| \| --- \| | |
| *alr2269* | alr2269-1 | ACTAAATCCGCAGCAACGAC | |
|  | alr2269-2 | CAGCACTTCAGCAGTTCTGG | |
|  | alr2269C-1 | CTCGAGTAACGGCCACGAGGATAG | |
|  | alr2269C-2 | AGGGTAAACACCCTGAAAATCCGT | |
| *all3983* | all3983-1 | GGTGTAAGGTTTGTCGCCAG | |
|  | all3983-2 | TGGCACAGGCTTAGGATTAG | |
|  | all3983-3 | GCGCCCTTGTAGCTGTTGCTG | |
|  | all3983-4 | CCAAGCTATTCCCCGCCAGC | |
| *alr2269* | alr2269-q-F | ggacaggatgtcaacgaacaaggac | |
|  | alr2269-q-R | ctgtcccgaatacgcgttgt | |
| *all3983* | all3983-q-F | gagcctaaccaagctattccc | |
|  | all3983-q-R | gccggcaatgttaacagcgt | |
| *rnpB* | rnpB-1 | gcgttggcggttgcagaccagt | |
|  | rnpB-2 | agtccccagtccccaatcttgg | |
| *alr2269* | gRNA-2269-F | AGATGTACAGTCAGCTATCAACAGCA | |
|  | gRNA-2269-R | AGACTGCTGTTGATAGCTGACTGTAC | |
|  | Cr-2269-F1 | ATGGCAGAAATTCGATATCTATGCTGCTGGTAGCTTATCTCAGCA | |
|  | Cr-2269-R1 | CGTAGCCGTTAACAGTTAAGCAACTAATACGGGAGATAAACGCATCGTC | |
|  | Cr-2269-F2 | GACGATGCGTTTATCTCCCGTATTAGTTGCTTAACTGTTAACGGCTACG | |
|  | Cr-2269-R2 | GCAACGTTGTTGCCATTGCGGCTCGGTAGTGAGTGACCAACT | |
|  | 2269-W-F | GACCGTTTCCGGCGCGACCTG | |
|  | 2269-W-R | CGAATACAGCCAGTATATAGAG | |
| *alr4893* | Cr-4893-F1 | TGGCAGAAATTCGATATCTACGGGACACATCACTCATAAGAAAC | |
|  | Cr-4893-R1 | GTACTCGACTTTCCCCTTGGTCGCTGGAGTTTGAGTTGTTGTCGCAG | |
| *alr4893* | Cr-4893-F2 | CTGCGACAACAACTCAAACTCCAGCGACCAAGGGGAAAGTCGAGTAC | |
|  | Cr-4893-R2 | GCAACGTTGTTGCCATTGCGCCTCATGTGCCACGGCAAAACG | |
|  | gRNA-4893-F | AGATATGGGTACGTTGAACTATCAAG | |
|  | gRNA-4893-R | AGACCTTGATAGTTCAACGTACCCAT | |
|  | 4893-W-F | CAATTGAACCCAACCGTCAAGG | |
|  | 4893-W-R | CGATTAACTACGGTCGCTGCC | |
| *alr0075* | Cr-0075-F1 | TGGCAGAAATTCGATATCTACAGTACGGACAGCACCAAACAAG | |
|  | Cr-0075-R1 | GCCAGTTGGTCATTTAAACCGCACGCATATTGGCAACCAATTAGTG | |
|  | Cr-0075-F2 | CACTAATTGGTTGCCAATATGCGTGCGGTTTAAATGACCAACTGGC | |
|  | Cr-0075-R2 | GCAACGTTGTTGCCATTGCGCATCTCCTCAATTAGCAACGCC | |
|  | gRNA-0075-F | AGATCAATAGTTGTGATTTTGGTGGT | |
|  | gRNA-0075-R | AGACACCACCAAAATCACAACTATTG | |
|  | 0075-W-F | CCTCCAATAGTTGCTTACACTC | |
|  | 0075-W-R | GCCCTAGACGATAAGGACTCG | |

*^a^* Ap, ampicillin; Cm, chloramphenicol; Em, erythromycin; Km, kanamycin; Nm, neomycin; Sm, streptomycin; Sp, spectinomycin; unless stated otherwise, the template for PCR reactions was *Anabaena* 7120 genomic DNA (https://www.ncbi.nlm.nih.gov/datasets/taxonomy/103690/).

*^b^* PCC, Pasteur Culture Collection.

*^c^* FACHB, Freshwater Algal Culture Collection of the Institute of Hydrobiology, Chinese Academy of Sciences.

**References**

Black TA, Cai Y, Wolk CP. 1993. Spatial expression and autoregulation of *hetR*, a gene involved in the control of heterocyst development in *Anabaena*. Mol Microbiol 9: 77-84.

Black TA, Wolk CP. 1994. Analysis of a Het- mutation in *Anabaena* sp. strain PCC 7120 implicates a secondary metabolite in the regulation of heterocyst spacing. J Bacteriol 176: 2282-2292.

Niu TC, Lin GM, Xie LR, Wang ZQ, Xing WY, Zhang JY, Zhang CC. 2019. Expanding the potential of CRISPR-Cpf1-based genome editing technology in the cyanobacterium *Anabaena* PCC 7120. ACS Synth Biol 8: 170-180.

Xu X, Kong R, Hu Y. 1993. High larvicidal activity of intact recombinant cyanobacterium *Anabaena* sp. PCC 7120 expressing Gene 51 and Gene 42 of *Bacillus sphaericus* 2297. FEMS Microbiol Lett 107: 247-250.

**Table S2 Comparative proteomic analysis of outer membrane proteins in the wild type (WT) and the *alr2269M* mutant of *Anabaena* sp. PCC 7120**

| **Proteins** | **alr2269M**  **-1** | **alr2269M**  **-2** | **alr2269M**  **-3** | **wt-1** | **wt-2** | **wt-3** | **alr2269M/wt**  **ratio** | **P value** | **β-barrel** | **Moslavac et al.** **†** | **Dong and Xu** ‡ |
| --- | --- | --- | --- | --- | --- | --- | --- | --- | --- | --- | --- |
| Alr0075 | 1.636 | 1.721 | 1.804 | 0.265 | 0.28 | 0.242 | 6.558 | 3.23164E-06 | * |  |  |
| Alr4893 | 1.454 | 1.379 | 1.485 | 0.2 | 0.2 | 0.282 | 6.331 | 9.06387E-05 | * |  |  |
| Alr3353 | 1.648 | 1.852 | 1.597 | 0.403 | 0.201 | 0.272 | 5.818 | 0.000952106 |  |  |  |
| All3983 | 1.504 | 1.494 | 1.548 | 0.268 | 0.275 | 0.265 | 5.626 | 3.84381E-08 |  | OM/Ext |  |
| Alr2231 | 0.262 | 0.313 | 4.472 | 0.395 | 0.234 | 0.366 | 5.072 | 0.442007413 | * |  |  |
| Alr2933 | 1.514 | 1.534 | 1.545 | 0.47 | 0.443 | 0.283 | 3.84 | 0.001028649 |  |  |  |
| All7611 | 1.602 | 1.528 | 1.486 | 0.504 | 0.5 | 0.452 | 3.17 | 9.78365E-06 |  | OM |  |
| Alr0702 | 1.482 | 1.432 | 1.473 | 0.474 | 0.484 | 0.535 | 2.938 | 9.97086E-06 |  | OM/PP |  |
| All7619 | 1.389 | 1.577 | 1.397 | 0.57 | 0.485 | 0.505 | 2.797 | 8.62594E-05 |  |  |  |
| All1776 | 1.426 | 1.408 | 1.47 | 0.499 | 0.526 | 0.548 | 2.736 | 4.67948E-06 |  | OM/Ext |  |
| Alr0092 | 1.404 | 1.414 | 1.36 | 0.594 | 0.576 | 0.518 | 2.475 | 3.02005E-05 |  | OM |  |
| All2610 | 1.399 | 1.43 | 1.382 | 0.585 | 0.606 | 0.536 | 2.438 | 1.91318E-05 | * |  |  |
| All0089 | 1.38 | 1.337 | 1.337 | 0.604 | 0.629 | 0.647 | 2.156 | 4.43839E-06 |  | OM/PP |  |
| All3118 | 1.379 | 1.317 | 1.326 | 0.549 | 0.762 | 0.683 | 2.017 | 0.001879208 |  |  |  |
| All2086 | 1.307 | 1.344 | 1.348 | 0.683 | 0.679 | 0.624 | 2.014 | 2.21135E-05 |  |  |  |
| All7319 | 1.351 | 1.157 | 1.443 | 0.51 | 0.698 | 0.86 | 1.911 | 0.015790849 |  |  |  |
| All3942 | 1.331 | 1.166 | 1.297 | 0.716 | 0.709 | 0.708 | 1.779 | 0.000145764 |  |  |  |
| All2674 | 1.26 | 1.224 | 1.242 | 0.717 | 0.698 | 0.683 | 1.776 | 3.91869E-06 | * |  |  |
| Alr3608 | 1.26 | 1.264 | 1.263 | 0.735 | 0.709 | 0.734 | 1.739 | 1.24708E-06 |  | OM |  |
| Alr2592 | 1.247 | 1.296 | 1.236 | 0.719 | 0.711 | 0.797 | 1.697 | 0.000172321 | * |  |  |
| All4539 | 1.272 | 1.263 | 1.24 | 0.746 | 0.783 | 0.771 | 1.641 | 6.74719E-06 |  |  |  |
| All1101 | 1.248 | 1.196 | 1.238 | 0.798 | 0.776 | 0.752 | 1.583 | 2.86344E-05 | * | OM |  |
| All7614 | 1.298 | 1.293 | 1.163 | 0.643 | 0.817 | 0.912 | 1.583 | 0.012796698 | * |  |  |
| All4388 | 1.186 | 1.168 | 1.224 | 0.769 | 0.8 | 0.75 | 1.543 | 4.90029E-05 |  | OM |  |
| All4999 | 1.236 | 1.2 | 1.192 | 0.794 | 0.812 | 0.747 | 1.542 | 9.20003E-05 |  | OM/Ext |  |
| Alr3243 | 1.408 | 1.165 | 1.014 | 0.817 | 0.862 | 0.784 | 1.456 | 0.020614133 |  |  |  |
| Alr2626 | 1.188 | 1.101 | 1.219 | 0.819 | 0.813 | 0.812 | 1.435 | 0.000300123 | * |  |  |
| Alr4550 | 1.228 | 1.155 | 1.126 | 0.728 | 0.837 | 0.932 | 1.405 | 0.010538252 | * | OM | ‡ |
| Alr0564 | 1.112 | 1.154 | 1.17 | 0.857 | 0.838 | 0.824 | 1.364 | 8.17156E-05 |  |  |  |
| All2236 | 1.245 | 1.047 | 1.179 | 0.874 | 0.863 | 0.81 | 1.363 | 0.005459584 | * |  |  |
| All0086 | 1.208 | 1.11 | 1.152 | 0.862 | 0.848 | 0.85 | 1.355 | 0.000264417 |  |  |  |
| Alr4147 | 1.142 | 1.152 | 1.092 | 0.85 | 0.841 | 0.877 | 1.319 | 0.000185439 |  |  |  |
| Alr2581 | 1.142 | 1.151 | 1.116 | 0.915 | 0.846 | 0.832 | 1.315 | 0.0008715 | * |  | ‡ |
| Alr2588 | 1.16 | 1.148 | 1.153 | 0.895 | 0.87 | 0.871 | 1.313 | 9.71514E-06 | * | OM |  |
| Alr1278 | 1.094 | 1.21 | 1.066 | 0.934 | 0.784 | 0.866 | 1.304 | 0.013888506 | * | OM |  |
| Alr3242 | 1.112 | 1.085 | 1.121 | 0.879 | 0.896 | 0.862 | 1.258 | 0.000102381 | * |  |  |
| Alr5340 | 1.138 | 1.126 | 1.094 | 0.928 | 0.853 | 0.889 | 1.258 | 0.00105137 |  |  |  |
| Alr2153 | 1.073 | 1.1 | 1.161 | 0.866 | 0.887 | 0.914 | 1.25 | 0.001351209 | * |  | ‡ |
| Alr1677 | 1.082 | 1.119 | 1.117 | 0.881 | 0.927 | 0.88 | 1.234 | 0.000489756 |  |  |  |
| Alr0140 | 1.099 | 1.105 | 1.093 | 0.91 | 0.936 | 0.909 | 1.197 | 5.82987E-05 |  |  |  |
| Alr3429 | 1.086 | 1.101 | 1.078 | 0.869 | 0.907 | 0.966 | 1.191 | 0.004999856 |  |  |  |
| Alr0397 | 1.042 | 1.076 | 1.12 | 0.958 | 0.872 | 0.924 | 1.176 | 0.009244108 | * | OM |  |
| Alr4579 | 1.037 | 1.063 | 1.056 | 0.951 | 0.938 | 0.898 | 1.132 | 0.002691184 |  |  |  |
| Alr4067 | 1.033 | 1.114 | 1.036 | 0.96 | 0.908 | 0.964 | 1.124 | 0.02046268 |  | OM |  |
| All2869 | 1.111 | 1.046 | 0.998 | 0.944 | 0.966 | 0.911 | 1.118 | 0.034839047 | * |  |  |
| All2148 | 1.08 | 1.031 | 0.997 | 0.945 | 0.876 | 1.09 | 1.068 | 0.367953872 | * |  |  |
| All2344 | 1.022 | 1.224 | 0.858 | 0.976 | 0.98 | 0.982 | 1.057 | 0.687028018 |  |  |  |
| All3286 | 0.634 | 1.517 | 0.732 | 0.881 | 0.97 | 0.883 | 1.054 | 0.936327568 |  |  |  |
| Alr3762 | 1.06 | 1.008 | 0.977 | 0.961 | 0.98 | 0.976 | 1.044 | 0.158303039 |  |  |  |
| Alr3546 | 1.003 | 1.013 | 1.05 | 1.012 | 0.941 | 0.986 | 1.043 | 0.169408216 |  |  |  |
| Alr3276 | 1.036 | 0.996 | 0.986 | 0.92 | 0.99 | 0.996 | 1.039 | 0.266597168 |  |  |  |
| Alr1666 | 1.039 | 0.936 | 1.067 | 1.016 | 0.931 | 0.991 | 1.035 | 0.516605743 |  | OM/PP |  |
| Alr2211 | 0.969 | 0.861 | 1.119 | 0.949 | 0.983 | 0.953 | 1.022 | 0.841819979 | * |  |  |
| All4499 | 1.092 | 1.008 | 0.919 | 0.826 | 0.908 | 1.227 | 1.02 | 0.819301289 | * | OM | ‡ |
| Alr0255 | 0.994 | 1.001 | 1.033 | 0.992 | 1.005 | 1.01 | 1.007 | 0.626016505 |  |  |  |
| All0927 | 0.909 | 1.088 | 0.97 | 0.934 | 1.06 | 1 | 0.991 | 0.877610528 |  |  |  |
| All1140 | 1.071 | 0.943 | 0.956 | 0.995 | 1.019 | 0.99 | 0.989 | 0.769568199 |  | OM |  |
| Alr2185 | 0.983 | 0.881 | 1.11 | 1.017 | 1.066 | 0.974 | 0.973 | 0.68469608 | * |  |  |
| Alr2887 | 0.966 | 0.946 | 1.036 | 0.998 | 0.8 | 1.259 | 0.964 | 0.888552827 | * | OM | ‡ |
| Alr0834 | 0.986 | 0.97 | 0.886 | 0.9 | 0.954 | 1.267 | 0.911 | 0.493977006 | * | OM | ‡ |
| All4294 | 0.929 | 0.968 | 0.966 | 1.112 | 1.041 | 1.057 | 0.892 | 0.008939194 |  | OM |  |
| All4401 | 0.916 | 0.949 | 0.958 | 1.003 | 1.096 | 1.12 | 0.877 | 0.023170322 |  |  |  |
| All3773 | 0.908 | 0.911 | 0.91 | 1.039 | 1.129 | 1.039 | 0.851 | 0.004402589 |  |  |  |
| Alr3884 | 0.875 | 0.952 | 0.934 | 1.097 | 1.103 | 1.095 | 0.838 | 0.002283048 |  |  |  |
| Alr4029 | 0.902 | 0.938 | 0.884 | 1.106 | 1.188 | 1.018 | 0.822 | 0.015531566 | * |  |  |
| Alr2596 | 0.879 | 0.955 | 0.898 | 1.265 | 1.068 | 1.09 | 0.798 | 0.019232897 | * |  |  |
| Alr1659 | 0.91 | 0.881 | 0.897 | 1.192 | 1.134 | 1.058 | 0.794 | 0.003066947 | * |  |  |
| All5036 | 0.857 | 0.935 | 0.875 | 1.059 | 1.184 | 1.137 | 0.789 | 0.004835249 |  |  |  |
| All2500 | 0.896 | 0.863 | 0.851 | 1.163 | 1.156 | 1.114 | 0.76 | 0.000178665 |  |  |  |
| Alr4028 | 0.882 | 0.907 | 0.812 | 1.147 | 1.159 | 1.162 | 0.75 | 0.00099056 | * |  |  |
| All0231 | 0.844 | 0.906 | 0.879 | 1.205 | 1.182 | 1.138 | 0.746 | 0.000380373 |  |  |  |
| All5116 | 0.811 | 0.83 | 0.832 | 1.193 | 1.205 | 1.125 | 0.702 | 0.000103212 | * |  |  |
| All3310 | 0.818 | 0.812 | 0.828 | 1.246 | 1.112 | 1.148 | 0.701 | 0.000500777 | * | OM |  |
| Alr2209 | 0.647 | 0.968 | 0.876 | 1.207 | 1.247 | 1.139 | 0.693 | 0.037551772 | * |  |  |
| All4924 | 0.799 | 0.817 | 0.816 | 1.216 | 1.254 | 1.142 | 0.673 | 0.000156563 | * |  |  |
| Alr2175 | 0.802 | 0.782 | 0.857 | 1.213 | 1.189 | 1.256 | 0.667 | 0.000215228 | * |  |  |
| Alr1690 | 0.788 | 0.781 | 0.776 | 1.126 | 1.342 | 1.247 | 0.631 | 0.000859281 |  | OM/PP |  |
| All1861 | 0.753 | 0.754 | 0.774 | 1.205 | 1.241 | 1.209 | 0.624 | 3.36461E-06 |  | OM |  |
| All4070 | 0.795 | 0.734 | 0.749 | 1.2 | 1.289 | 1.277 | 0.605 | 0.000106124 |  |  |  |
| All0493 | 0.766 | 0.749 | 0.742 | 1.252 | 1.307 | 1.198 | 0.601 | 4.562E-05 |  |  |  |
| Alr1819 | 0.78 | 0.714 | 0.726 | 1.235 | 1.222 | 1.254 | 0.598 | 5.26552E-05 | * | OM |  |
| All0443 | 0.769 | 0.757 | 0.715 | 1.255 | 1.31 | 1.28 | 0.583 | 2.87019E-05 |  |  |  |
| Alr1031 | 0.595 | 0.983 | 0.568 | 1.312 | 1.356 | 1.229 | 0.551 | 0.024245478 | * |  |  |
| Alr4741 | 0.643 | 0.692 | 0.741 | 1.244 | 1.139 | 1.398 | 0.549 | 0.0011562 | * |  |  |
| Alr4740 | 0.623 | 0.697 | 0.744 | 1.216 | 1.026 | 1.535 | 0.546 | 0.00970986 |  | OM |  |
| All4090 | 0.661 | 0.786 | 0.643 | 1.371 | 1.344 | 1.22 | 0.531 | 0.00093121 |  |  |  |
| All5163 | 0.681 | 0.716 | 0.68 | 1.207 | 1.603 | 1.168 | 0.522 | 0.003289013 |  |  |  |
| All5100 | 0.66 | 0.702 | 0.684 | 1.382 | 1.28 | 1.314 | 0.515 | 2.07742E-05 |  |  |  |
| All4026 | 0.666 | 0.665 | 0.655 | 1.414 | 1.227 | 1.317 | 0.502 | 7.60345E-05 | * | OM |  |
| Alr3345 | 0.598 | 0.648 | 0.658 | 1.418 | 1.01 | 1.485 | 0.487 | 0.004826516 |  |  |  |
| Alr0093 | 0.605 | 0.676 | 0.626 | 1.415 | 1.362 | 1.445 | 0.452 | 2.79775E-05 |  |  |  |
| Alr2117 | 0.567 | 0.615 | 0.637 | 1.412 | 1.262 | 1.44 | 0.442 | 0.000109112 |  |  |  |
| Alr5178 | 0.532 | 0.556 | 0.592 | 1.467 | 1.302 | 1.472 | 0.396 | 5.37821E-05 |  |  |  |
| Alr7326 | 0.49 | 0.554 | 0.552 | 1.37 | 1.412 | 1.46 | 0.376 | 2.48788E-05 |  | OM/Ext |  |
| All7072 | 0.418 | 0.642 | 0.587 | 1.402 | 1.41 | 1.602 | 0.373 | 0.001909855 |  |  |  |
| All5316 | 0.5 | 0.532 | 0.474 | 1.395 | 1.448 | 1.507 | 0.346 | 1.21371E-05 |  |  |  |
| All2430 | 0.405 | 0.481 | 0.536 | 1.519 | 1.537 | 1.503 | 0.312 | 0.0001384 |  | OM |  |
| All4130 | 0.46 | 0.474 | 0.419 | 1.482 | 1.505 | 1.479 | 0.303 | 5.79729E-06 |  |  |  |
| All8079 | 0.438 | 0.42 | 0.351 | 1.593 | 1.572 | 1.565 | 0.256 | 3.62113E-05 |  |  |  |
| All0462 | 0.334 | 0.316 | 0.356 | 1.495 | 1.543 | 1.523 | 0.221 | 1.84112E-06 |  |  |  |
| All0405 | 0.378 | 0.343 | 0.32 | 1.65 | 1.548 | 1.587 | 0.218 | 7.85043E-06 |  | OM/PP |  |
| Alr2269 | 0.222 | 0.297 | 0.285 | 1.571 | 1.523 | 1.548 | 0.173** | 4.2687E-05 | * | OM | ‡ |

***** indicates proteins with the β-barrel structure.

**†** OM, outer membrane; Ext, extracellular; PP, periplasmic space. Moslavac et al, 2005. J Proteome Res 4: 1330-1338.

**Alr2269 peptides were still detectable in the *alr2269M* strain because the insertion was in the middle of *alr2269*.

‡ indicates proteins detected in 2-D gel analysis. Dong and Xu, 2009. Acta Hydrobiol Sin 33: 994-997.

**Table S3 Parameters of quantitative polymerase chain reactions (qPCR)**

| Parameter  Primer | Amplification efficiency* | Standard curve linearity R^2^ | Melting temperature (Tm)** |
| --- | --- | --- | --- |
| rnpB-1 | 92.90% | 0.993 | Single peak  83.5 ℃ |
| rnpB-2 |  |  |  |
| alr2269-q-F | 92.20% | 0.994 | Single peak  85.5 ℃ |
| alr2269-q-R |  |  |  |
| all3983-q-F | 96.10% | 0.994 | Single peak  85.0 ℃ |
| all3983-q-R |  |  |  |

* A difference of <5% in amplification efficiency between the target gene and reference gene is a prerequisite for relative quantification using the 2^−ΔΔ^*^C^*^T^ method.

** A single peak in the melting curve indicates specific amplification without primer dimers and non-specific products.

**Table S4 Threshold cycle (C_t_) *^a^* values for qPCR analysis of *alr2269*** **and calculations of 2^-△△^*^Ct^***

| *alr2269M* | | | | | | | |
| --- | --- | --- | --- | --- | --- | --- | --- |
| *alr2269* or *rnpB* | | Biological replicates *^b^* | | | | | |
| *alr2269* | | 1 | 2 | 3 | 4 | 5 | 6 |
| Technical replicates *^c^* | 1 | 32.63 | 39.99 *^d^* | 32.81 | 33.10 | 32.86 | 33.25 |
|  | 2 | 32.92 | 32.43 | 33.03 | 33.35 | 31.72 | 33.02 |
|  | 3 | 33.12 | 32.85 | 33.51 | 32.68 | 32.58 | 33.27 |
| Mean ± SD | | 32.89 ± 0.25 | 32.64 ± 0.30 | 33.12 ± 0.36 | 33.04 ± 0.34 | 32.39 ± 0.59 | 33.18 ± 0.14 |
| *rnpB* | | 1 | 2 | 3 | 4 | 5 | 6 |
| Technical replicates | 1 | 20.72 | 20.99 | 20.88 | 21.01 | 20.78 | 20.81 |
|  | 2 | 20.78 | 20.95 | 21.01 | 20.93 | 21.38 | 20.80 |
|  | 3 | 20.81 | 21.01 | 20.85 | 21.09 | N/A *^e^* | 21.06 |
| Mean ± SD | | 20.77 ± 0.05 | 20.98 ± 0.03 | 20.91 ± 0.09 | 21.01 ± 0.08 | 21.08 ± 0.42 | 20.89 ± 0.15 |
| WT | | | | | | | |
| *alr2269* or *rnpB* | | Biological replicates | | | | | |
| *alr2269* | | 1 | 2 | 3 | 4 | 5 | 6 |
| Technical replicates | 1 | 20.29 | 20.24 | 20.16 | 20.46 | 20.50 | 20.80 |
|  | 2 | 20.11 | 20.33 | 20.17 | 20.72 | 20.38 | 20.71 |
|  | 3 | 20.07 | 20.25 | 20.16 | 20.52 | 20.57 | 22.53 |
| Mean ± SD | | 20.16 ± 0.12 | 20.27 ± 0.05 | 20.16 ± 0.01 | 20.57 ± 0.14 | 20.48 ± 0.10 | 21.35 ± 1.03 |
| *rnpB* | | 1 | 2 | 3 | 4 | 5 | 6 |
| Technical replicates | 1 | 20.35 | 20.64 | 20.29 | 20.82 | 20.66 | 21.14 |
|  | 2 | 20.36 | 20.54 | 20.34 | 20.89 | 20.72 | 21.06 |
|  | 3 | 20.29 | 20.45 | 20.31 | 20.86 | 20.66 | 21.14 |
| Mean ± SD | | 20.33 ± 0.04 | 20.54 ± 0.10 | 20.31 ± 0.03 | 20.86 ± 0.04 | 20.68 ± 0.03 | 21.11 ± 0.05 |
| Calculations of 2^-△△^*^Ct^* | | | | | | | |
| *alr2269M* △C_t_ | | 12.12 | 11.66 | 12.20 | 12.03 | 11.31 | 12.29 |
| WT △C_t_ | | -0.18 | -0.27 | -0.15 | -0.29 | -0.20 | 0.23 |
| WT △C_t_ Mean | | -0.14 | | | | | |
| *alr2269* △△C_t_ | | 12.26 | 11.80 | 12.35 | 12.18 | 11.45 | 12.43 |
| WT 2^-△△^*^Ct^* | | 1.13027 | 1.20581 | 1.10957 | 1.22264 | 1.14605 | 0.85067 |
| *alr2269M* 2^-△△^*^Ct^* | | 0.00020 | 0.00028 | 0.00019 | 0.00022 | 0.00036 | 0.00018 |

*^a^* C_t_ value (Threshold cycle): The PCR cycle number at which the fluorescence intensity in the reaction tube exceeds a predefined threshold for the first time during the qPCR amplification.

*^b^* Biological replicates mean independent samples.

*^c^* Technical replicates mean PCR replicates per biological sample.

*^d^* This C_t_ value for *alr2269M* was significantly higher than other replicates and close to the detection limit, thus excluded from the analysis.

*^e^* This replicate showed no detectable reading and was therefore excluded; values from the other two replicates were used for the analysis.

**Table S5** **Threshold cycle values for qPCR analysis of *all3983* and calculations of 2^-△△^*^Ct^***

| *all3983M* | | | | | | | |
| --- | --- | --- | --- | --- | --- | --- | --- |
| *all3983* or *rnpB* | | Biological replicates | | | | | |
| *all3983* | | 1 | 2 | 3 | 4 | 5 | 6 |
| Technical replicates | 1 | 33.02 | 33.03 | 32.52 | 32.34 | 31.59 | 30.88 |
|  | 2 | 32.74 | 32.76 | 32.16 | 33.74 | 32.41 | 31.90 |
|  | 3 | 33.08 | 32.52 | 33.12 | 32.54 | 32.24 | 32.84 |
| Mean ± SD | | 32.95 ± 0.18 | 32.77 ± 0.26 | 32.60 ± 0.48 | 32.87 ± 0.76 | 32.08 ± 0.43 | 31.87 ± 0.98 |
| *rnpB* | | 1 | 2 | 3 | 4 | 5 | 6 |
| Technical replicates | 1 | 21.12 | 21.42 | 20.13 | 21.31 | 19.08 | 19.37 |
|  | 2 | 21.07 | 21.42 | 20.87 | 21.27 | 19.40 | 19.56 |
|  | 3 | 21.10 | 21.51 | 20.70 | 21.26 | 19.43 | 19.64 |
| Mean ± SD | | 21.10 ± 0.03 | 21.45 ± 0.05 | 20.57 ± 0.39 | 21.28 ± 0.03 | 19.30 ± 0.19 | 19.52 ± 0.14 |
| WT | | | | | | | |
| *all3983* or *rnpB* | | Biological replicates | | | | | |
| *all3983* | | 1 | 2 | 3 | 4 | 5 | 6 |
| Technical replicates | 1 | 20.29 | 20.23 | 20.36 | 20.51 | 20.67 | 20.59 |
|  | 2 | 20.31 | 20.28 | 20.31 | 20.44 | N/A | N/A |
|  | 3 | 20.39 | 20.29 | 20.46 | 20.50 | 20.80 | 20.61 |
| Mean ± SD | | 20.33 ± 0.05 | 20.27 ± 0.03 | 20.38 ± 0.08 | 20.48 ± 0.04 | 20.74 ± 0.09 | 20.60 ± 0.01 |
| *rnpB* | | 1 | 2 | 3 | 4 | 5 | 6 |
| Technical replicates | 1 | 20.88 | 20.63 | 20.80 | 20.96 | 21.06 | 21.04 |
|  | 2 | 20.69 | 20.71 | 20.76 | 21.02 | 21.12 | 21.03 |
|  | 3 | 20.73 | 20.72 | 20.84 | 20.99 | 21.07 | 21.14 |
| Mean ± SD | | 20.77 ± 0.10 | 20.69 ± 0.05 | 20.80 ± 0.04 | 20.99 ± 0.03 | 21.08 ± 0.03 | 21.07 ± 0.06 |
| Calculations of 2^-△△^*^Ct^* | | | | | | | |
| *all3983M* △C_t_ | | 11.85 | 11.32 | 12.03 | 11.59 | 12.78 | 12.35 |
| WT △C_t_ | | -0.44 | -0.42 | -0.42 | -0.51 | -0.34 | -0.47 |
| WT △C_t_ Mean | | -0.43 | | | | | |
| *all3983M* △△C_t_ | | 12.28 | 11.75 | 12.47 | 12.02 | 13.21 | 12.78 |
| WT 2^-△△^*^Ct^* | | 1.35347 | 1.33793 | 1.34102 | 1.42076 | 1.27309 | 1.38511 |
| *all3983M* 2^-△△^*^Ct^* | | 0.00020 | 0.00029 | 0.00018 | 0.00024 | 0.00011 | 0.00014 |
